# Synthesis of Large-Scale Monolayer 1T′-MoTe_2_ and Its Stabilization *via* Scalable hBN Encapsulation

**DOI:** 10.1021/acsnano.0c05936

**Published:** 2021-02-19

**Authors:** Simona Pace, Leonardo Martini, Domenica Convertino, Dong Hoon Keum, Stiven Forti, Sergio Pezzini, Filippo Fabbri, Vaidotas Mišeikis, Camilla Coletti

**Affiliations:** † Center for Nanotechnology Innovation @NEST, Istituto Italiano di Tecnologia, Piazza San Silvestro 12, 56127 Pisa, Italy; ‡ Graphene Laboratories, Istituto Italiano di Tecnologia, Via Morego 30, 16163 Genova, Italy

**Keywords:** 1T′ molybdenum ditelluride, chemical vapor deposition, environmental stability, hBN encapsulation, large area

## Abstract

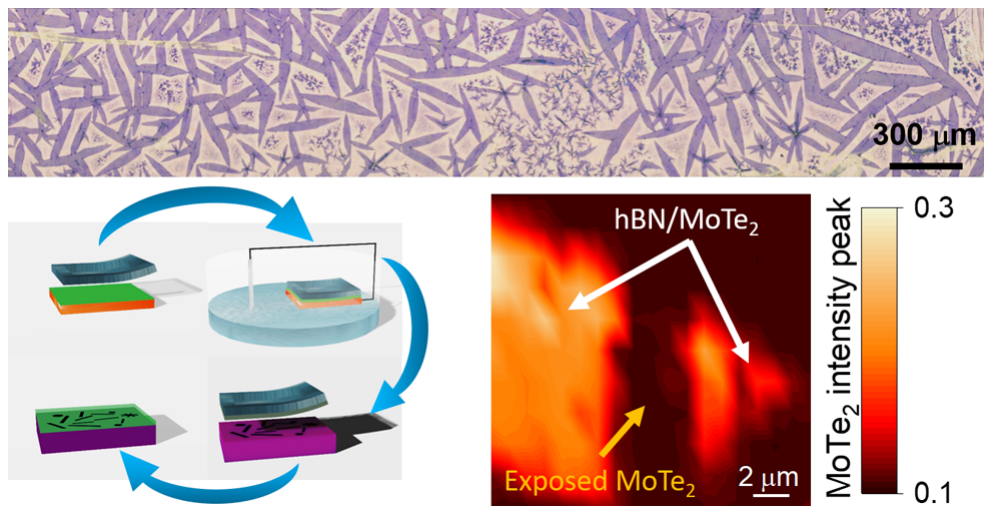

Out of the different structural phases of molybdenum ditelluride (MoTe_2_), the distorted octahedral 1T′ possesses great interest for fundamental physics and is a promising candidate for the implementation of innovative devices such as topological transistors. Indeed, 1T′-MoTe_2_ is a semimetal with superconductivity, which has been predicted to be a Weyl semimetal and a quantum spin Hall insulator in bulk and monolayer form, respectively. Large instability of monolayer 1T′-MoTe_2_ in environmental conditions, however, has made its investigation extremely challenging so far. In this work, we demonstrate homogeneous growth of large single-crystal (up to 500 μm) monolayer 1T′-MoTe_2_*via* chemical vapor deposition (CVD) and its stabilization in air with a scalable encapsulation approach. The encapsulant is obtained by electrochemically delaminating CVD hexagonal boron nitride (hBN) from copper foil, and it is applied on the freshly grown 1T′-MoTe_2_*via* a top-down dry lamination step. The structural and electrical properties of encapsulated 1T′-MoTe_2_ have been monitored over several months to assess the degree of degradation of the material. We find that when encapsulated with hBN, the lifetime of monolayer 1T′-MoTe_2_ successfully increases from a few minutes to more than a month. Furthermore, the encapsulated monolayer can be subjected to transfer, device processing, and heating and cooling cycles without degradation of its properties. The potential of this scalable heterostack is confirmed by the observation of signatures of low-temperature phase transition in monolayer 1T′-MoTe_2_ by both Raman spectroscopy and electrical measurements. The growth and encapsulation methods reported in this work can be employed for further fundamental studies of this enticing material as well as facilitate the technological development of monolayer 1T′-MoTe_2_.

Transition metal dichalcogenides (TMDs) have recently attracted large interest due to their wide range of electronic properties. Depending on the nature of the chalcogenide and the transition metal, semiconductor, semimetal, or topological insulator behavior can be observed,^1^ making this class of materials an enticing platform for several fields of applications, such as electronics,^2,3^ spintronics,^4^ and optoelectronics.^5,6^

Within such a class, MoTe_2_ has received increasing attention over the past few years, since it displays two thermodynamically stable polymorphs with peculiar electronic and structural properties. Similarly to other TMDs, bulk MoTe_2_ is stable in the 2H semiconducting phase and shows an indirect-to-direct band gap transition when thinned down to the limit of bi- or monolayer.^7,8^ 2H-MoTe_2_ has a near-infrared band gap (about 1 eV) and shows mobility up to 137 cm^2^V^-1^ s^-1^ at 77 K,^9^ strong spin–orbit coupling,^10^ valley degree of freedom,^11^ and ambipolar behavior,^12^ which make it a promising candidate for spintronic, valleytronic, electronic, and near-infrared optoelectronic applications. In the second metastable polymorph, *i.e*., 1T′-MoTe_2_, Mo is in the center of a distorted octahedron of Te atoms, resulting in a monoclinic structure or distorted 1T-MoTe_2_.^13^ 1T′-MoTe_2_ is a semimetal^14^ that exhibits large magnetoresistance^15^ and superconductivity.^16^ 1T′-MoTe_2_ is also expected to be a type-II Weyl semimetal in bulk form^17^ and, as a monolayer, a 2D topological and large-gap quantum spin Hall (QSH) insulator,^18^ properties that can be exploited in spintronic and quantum computational applications. Differently from the sulfide and selenide counterparts, in MoTe_2_ the energy gap between the stable 2H and the metastable 1T′ phases is only 25 meV,^13,19^ resulting in reversible phase transition when it is subjected to strain,^20^ high temperature,^21^ or laser exposure.^13^ The possibility to pattern the phase of MoTe_2_ in a deterministic manner gives way to interesting opportunities in terms of both low-resistance contacts and phase-change memory.^22,23^ Moreover, while the synthesis of monolayer 1T′-MoS_2_ and 1T′-MoSe_2_ is extremely challenging,^24^ monolayer 1T′-MoTe_2_ can be directly grown *via* chemical vapor deposition (CVD) thanks to the relative stability of the distorted 1T phase in telluride-based TMDs. If single crystals of MoTe_2_ are grown *via* CVD, the 1T′-MoTe_2_ phase can be distinguished from the semiconducting 2H-MoTe_2_ due to the different shapes in which the two phases tend to naturally grow. While 2H-MoTe_2_ is stable in a hexagonal phase (space group *P*6_3_/*mmc*^25^) and hence grows with a hexagonal symmetry, 1T′-MoTe_2_ is stable in a monoclinic structure (space group *P*2_1_/*m*^26^). Therefore, single crystals of 1T′-MoTe_2_ tend to grow in an elongated shape, following the symmetry of its crystal structure.^19^ To date, the CVD growth of single-crystal monolayer 1T′-MoTe_2_ flakes with a lateral size up to 200 μm has been demonstrated.^27^

Despite the large spectrum of enticing perspectives, the experimental investigation of this material has been so far challenged by the high reactivity of MoTe_2_ when exposed to air. Indeed, due to the small difference between tellurium and molybdenum electronegativity, which results in a weak covalent Mo–Te bond, monolayer MoTe_2_ tends to react with environmental oxygen and to fully degrade in the first week when in the 2H phase^28,29^ and within a few hours in the 1T′ phase.^30^ Few-layer 1T′-MoTe_2_ tends to oxidize in a self-limited process, which generates a homogeneous oxide film on the top of the underlying intact layers of 1T′-MoTe_2_.^31^ These different reactivities have allowed, to date, a number of fundamental and applicative studies to be performed for monolayer 2H-MoTe_2_^7,28,32−36^ and few-layer 1T′-MoTe_2_,^14,31,37−40^ while only a few works have investigated the properties of monolayer 1T′-MoTe_2_.^27,30,41,42^

The high reactivity of monolayer 1T′-MoTe_2_ is indeed incompatible with most of characterization techniques, regardless of the quality of the starting material. Moreover, the final quality of devices based on monolayer 1T′-MoTe_2_ is expected to be much lower than that of the starting material, due to the considerable time required for device fabrication. For this reason, many properties of this class of TMDs remain yet unexplored, and a reliable and scalable encapsulation method is therefore necessary to exploit the full potential of monolayer 1T′-MoTe_2_ for fundamental studies and technological applications.

A few works^41,43^ have already used graphene-based encapsulation in order to prevent the interaction between environmental oxygen and 1T′-MoTe_2_ and characterized the properties of the material before degradation. This semimetal–semimetal interface can be interesting for fundamental studies, such as spin–galvanic-based applications;^37,44−46^ however, several technological applications will require insulating–semimetal heterostructures. Other TMDs are often encapsulated using insulating thin films directly deposited on the TMD material *via* atomic layer deposition (ALD) or physical vapor deposition (PVD).^47−49^ However, the reactive environment required for the deposition is expected to be incompatible with monolayer 1T′-MoTe_2_. Hexagonal boron nitride (hBN), on the other hand, is an inert layered material that is widely used as encapsulant for TMD- and graphene-based devices,^50−52^ due to its insulating behavior (band gap 5.97 eV) and chemical stability.^53^ Bulk flakes of hBN have also been employed to encapsulate exfoliated few-layer 2H-MoTe_2_^13,54^ and multilayer 1T′-MoTe_2_,^55^ leading to improved stability and promising results in nanoelectronics^56^ and optoelectronics.^57^ It should be noted that to date no work has reported encapsulation of monolayer 1T′-MoTe_2_ with hBN (either exfoliated or CVD), due to the difficulty of developing a rapid and clean approach that allows maintaining the material properties. This hurdle is strongly hindering fundamental studies on this enticing material.

In this work, we report on the CVD growth of monolayer 1T′-MoTe_2_ with a lateral size up to 500 μm and its scalable encapsulation *via* semidry transfer of few-layer CVD hBN. To the best of our knowledge, there is no other report on reliable, fully scalable growth and insulating encapsulation demonstrated for large-area, monolayer 1T′-MoTe_2_. Large-scale monolayer crystals are grown homogeneously over SiO_2_ substrates *via* liquid-precursor CVD. Detached CVD-hBN membranes, obtained *via* electrochemical delamination from the growth substrate, are then used to encapsulate the 1T′-MoTe_2_ right after the growth. With this approach, air stability of the material is increased from a few minutes to more than a month, a device operating through tunneling contacts can be fabricated, and fundamental properties such as phase transition are finally accessible.

## Results and Discussion

### Growth of Large Single-Crystal 1T′-MoTe_2_

**Figure 1:**
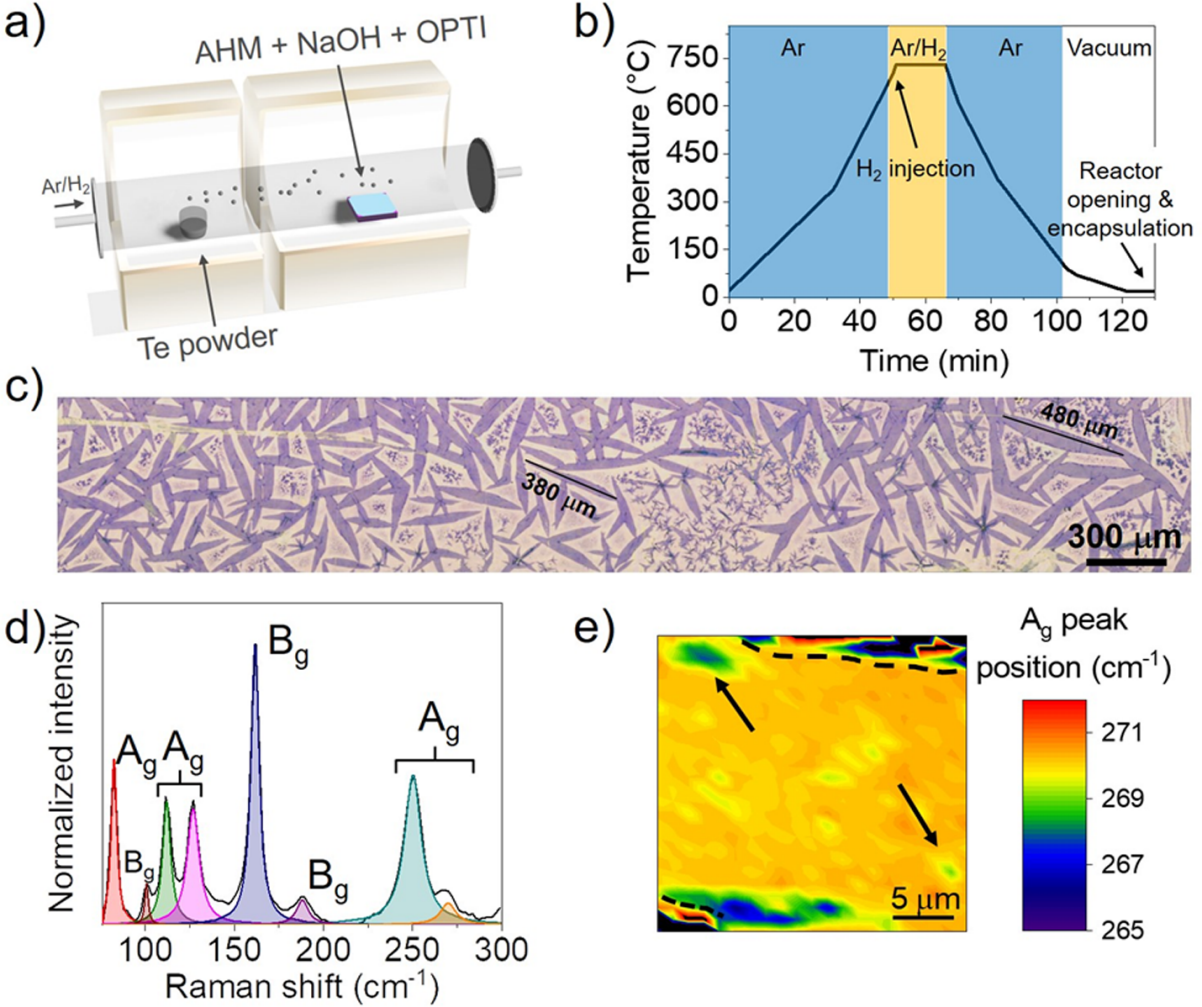
(a) Graphical representation of the reactor for the growth of 1T′-MoTe_2_. (b) Schematic plot showing the main steps of 1T′-MoTe_2_ growth using LqP-CVD. (c) Optical image of as-grown monolayer 1T′-MoTe_2_ single crystals on a SiO_2_ growth substrate. From optical contrast, the crystals are mostly monolayer with an average size of about 250 × 30 μm^2^. (d) Representative Raman spectrum of monolayer 1T′-MoTe_2_ obtained using a 532 nm laser. The fitted A_g_ and B_g_ peaks are highlighted. (e) Raman map showing the peak position of the thickness-dependent A_g_ peak at about 269 cm^–1^; the edges of the crystal are highlighted by the black dashed lines. The crystal shows homogeneous monolayer thickness on the entire area with isolated bilayer islands (black arrows).

Monolayer 1T′-MoTe_2_ reported in this work has been grown *via* liquid-precursor chemical vapor deposition (LqP-CVD),^58^ in which the transition metal precursor is dissolved in an aqueous solution and spun directly on the growth substrate. Compared to other growth techniques, the main advantages of LqP-CVD are its relatively low cost (only a quartz tube is needed), the adoption of precursors with low toxicity, and the ease of transfer of the grown material. With respect to the process described in ref (30), we also introduce a substrate preparation step that allows obtaining a homogeneous coverage of large single crystals. Specifically, oxygen plasma is first performed on the SiO_2_ substrate before the growth to enhance and make uniform the hydrophilicity of the growth substrate and, in turn, the distribution of the reactants on the surface. Subsequently, an aqueous solution containing ammonium heptamolybdate tetrahydrate (AHM), as Mo precursor, NaOH, as growth promoter, and commercial Opti Prep (OPTI), as density gradient medium, is prepared and spun directly on the SiO_2_ substrate, while tellurium powder is used as chalcogenide source (see Methods section for further details). The growth then takes place in a horizontal hot-wall reactor (Figure 1a) at 730 °C, near atmospheric pressure and under a reactive mix of 3% hydrogen in argon gas flux (Figure 1b). Upon optimization of the AHM:NaOH:OPTI solutions ratio (1:1:0.5) and growth conditions, a homogeneous coverage of monolayer 1T′-MoTe_2_ single crystals with an average size of 250 μm × 30 μm (and record size of 480 μm × 65 μm) is obtained on the SiO_2_ substrate, as shown in Figure 1c. Different crystal size and sample coverage can also be obtained by carefully choosing the speed at which the spinning step is carried out. While in this work 2900 rpm is chosen as spinning speed in order to obtain large area, yet isolated, single crystals, higher coverage and crystal size can be obtained by lowering this speed. If the spinning speed is lowered down to 1900 rpm, while all other growth parameters are maintained the same, full coverage can be obtained with a level of monolayer coverage of about 80% (Figure S1c). It is also worth noting that if the growth solution is spun homogeneously on the surface of the substrate, monolayer 1T′-MoTe_2_ single crystals are obtained on the entire substrate surface, suggesting that the only limitation to the further scalability of this synthesis method is the selection of the quartz tube in the reactor. The importance of the oxygen plasma step is evident in Figure S1, in which the same growth solution and conditions are used on both nontreated and plasma-treated substrates. Smaller and less homogeneous crystals (*i.e*., with a high incidence of bulk inclusions) are visible on nontreated substrates, while larger and homogeneous monolayer crystals are observed on the plasma-treated ones. The pretreatment of the substrate and the use of a density gradient (OptiPrep) are indeed necessary to achieve a uniform distribution of precursor (AHM) and promoter (NaOH) on the surface of the substrate before the growth, so that the nominal ratio in the growth solution is obtained uniformly on the entire surface. Specifically, the oxygen plasma step is instrumental to enhance the hydrophilicity of the SiO_2_ surface and to reduce the amount of the density gradient medium needed to obtain a homogeneous solution spinning. Hence, using this additional step, large monolayer 1T′-MoTe_2_ single crystals are obtained, while avoiding three-dimensional growth, which might be favored by accumulation of OptiPrep during the growth that was not burnt away. Due to the reactivity of the material under study, a freshly grown sample was used to obtain Raman data, and a representative spectrum is displayed in Figure 1d. The characteristic peaks of the 1T′ phase are visible at 84, 102, 113, 128, 163, 188, 252, and 269 cm^–1^,^40^ which are in good agreement with the Raman shifts predicted from theoretical calculations.^59^ To confirm the single-crystal nature of the elongated 1T′-MoTe_2_ flakes visible *via* optical microscopy, polarized Raman spectroscopy was carried out. The Raman maps of the 250 cm^–1^ A_g_ peak, obtained with the analyzer and detector either both perpendicular or both parallel to the [100] direction, for two misoriented flakes are reported in Figure S2. Homogeneous intensity of the Raman peak within each crystal indicates that the 1T′-MoTe_2_ flakes grown using LqCVD are single-crystal.^60^ In agreement with previous works on 1T′-MoTe_2_, the monolayer nature of the material reported here is confirmed by the position of the A_g_ Raman peak at 269 cm^–1^. The out-of-plane A_g_ vibrational mode centered at 269 cm^–1^ is indeed known to be sensitive to the number of layers of the material, and a red-shift from 269 cm^–1^ to 265 cm^–1^ and then 258 cm^–1^ is expected when the MoTe_2_ thickness increases from monolayer to few-layer to bulk, respectively.^14^ To assess the homogeneity of the MoTe_2_ single crystals, we mapped the position of the Raman peak at 269 cm^–1^ over a 25 × 25 μm^2^ area, as reported in Figure 1e. From Raman mapping results, the single crystal is confirmed to be a homogeneous monolayer.^41^ For the sake of comparison, an optical image and a Raman spectrum of bulk 1T′-MoTe_2_ are reported in Figure S3, in which a clear red-shift of the A_g_ peak position from 269 cm^–1^ to about 260 cm^–1^ is visible. Atomic force microscopy (AFM) measurements further confirm the monolayer nature of the material, as its thickness is found to be 0.82 ± 0.04 nm (Figure S4). Since it has been observed that the roughness of 1T′-MoTe_2_ might change during the degradation in environmental conditions,^30^ AFM images were obtained on 1T′-MoTe_2_ encapsulated in hBN, employing the method discussed in the next section. Finally, in Figure 1e, two bilayer islands are also observed along the edges of the crystal (black arrows). Similar results were already observed in monolayer MoTe_2_ and WTe_2_ single crystals grown using CVD, in which few-layer patches often accumulate along the edges of the single crystal^27,61^ or at the boundary of star-shaped crystals^41,62^ (see also Figure 3a–d). We speculate that these patches might be the result of the higher reactivity of edges and grain boundaries, where residual precursors can accumulate and react during the cooling after the growth.

**Figure 2:**
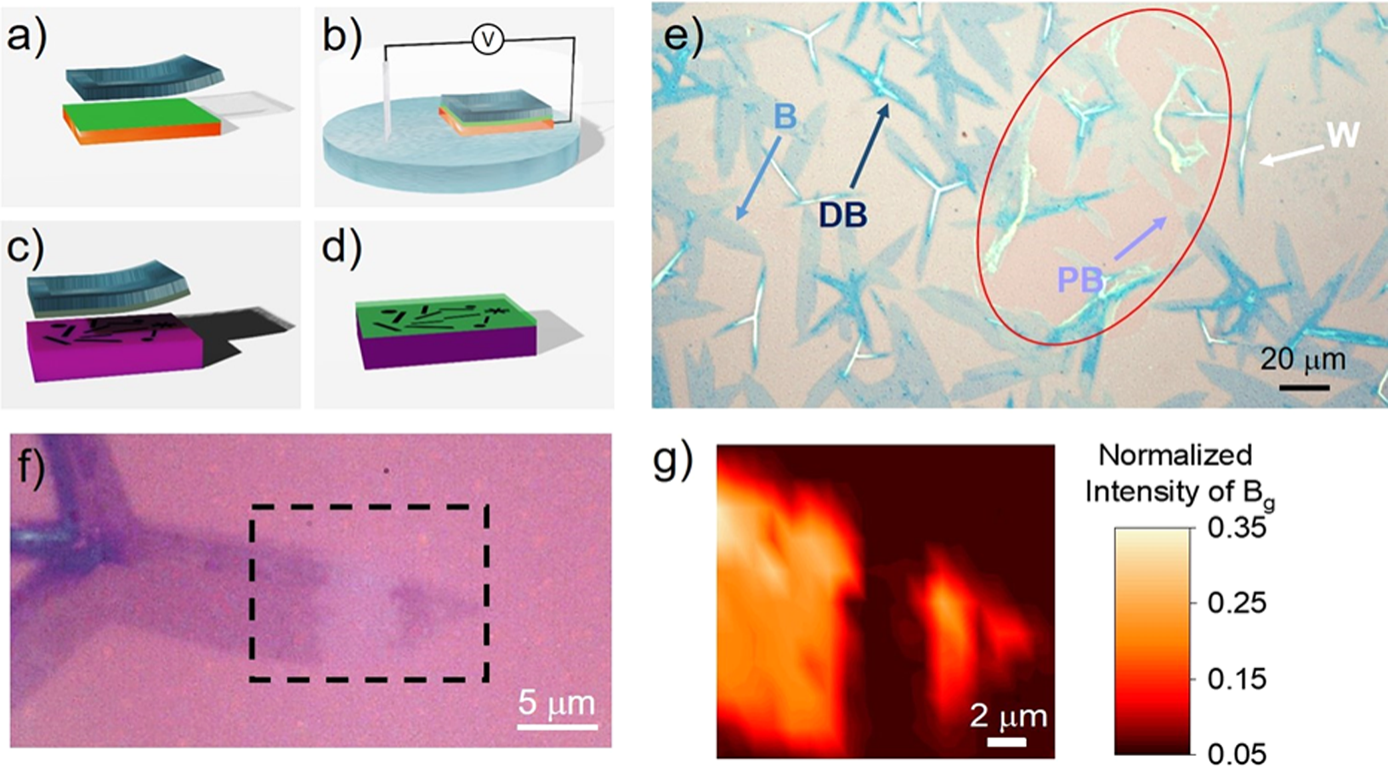
(a–d) Schematic representation of the main steps of the encapsulation method: the CVD hBN on copper foil is first covered by a double polymeric membrane and a PDMS frame a few millimeters thick (a); the copper foil is then immersed in a NaOH solution, where a constant voltage is applied until complete delamination (b); the free-standing membrane is then laminated on the freshly grown 1T′-MoTe_2_ sample in a top-down, fully dry fashion (c); the encapsulated sample is then cleaned in acetone and isopropanol (d). (e) Optical image of 1T′-MoTe_2_ covered by hBN 1 week after the encapsulation. Four different contrasts are visible: white (W) and dark blue (DB) for bulk 1T′-MoTe_2_, blue (B) for monolayer 1T′-MoTe_2_, and pale blue (PB) for oxidized monolayer 1T′-MoTe_2_. The air bubble beneath the hBN and the exposed 1T′-MoTe_2_ is highlighted by the red circle. (f) Optical image of an encapsulated crystal 1 month after hBN encapsulation. An hBN tear is visible within the black square. (g) Normalized intensity Raman maps of the B_g_ peak at 163 cm^–1^ taken on the area highlighted by the black square in panel f. A clear step in the intensity is visible in panel g, in good agreement with the different contrast in the optical image (f), confirming the high level of tightness of the encapsulation.

**Figure 3:**
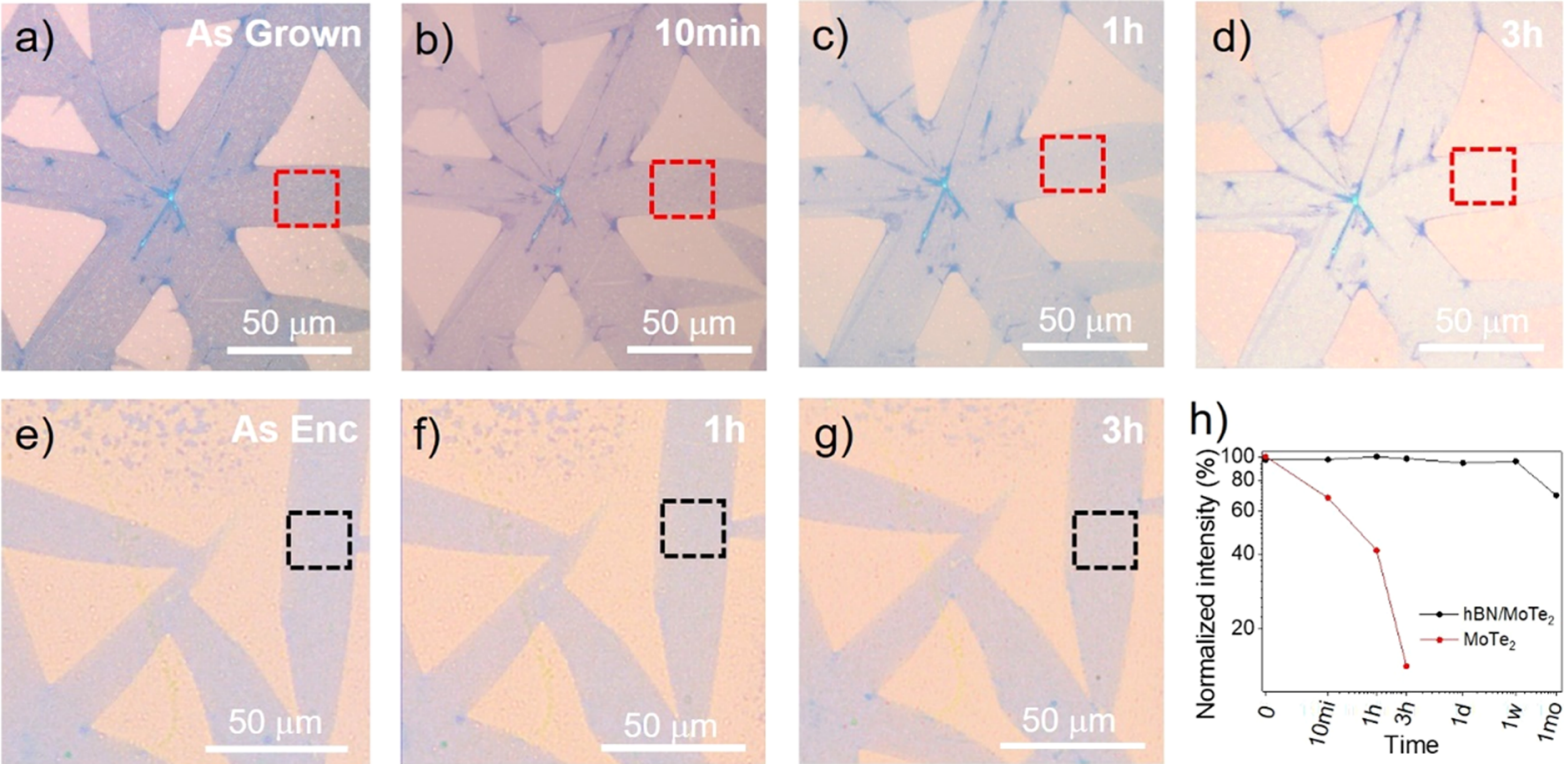
(a–d) Optical images of exposed 1T′-MoTe_2_ right after the growth (a) and after 10 min (b), 1 h (c), and 3 h (d). A clear dimming of the contrast from blue (B) to pale blue (PB) is visible. (e–g) Optical images of encapsulated 1T-MoTe_2_ right after the encapsulation (e) and after 1 h (f) and 3 h (g). No clear change of contrast is visible. (h) Trend over time of the normalized contrast intensity of both exposed and encapsulated sample extrapolated from the area highlighted by red and black squares respectively (a–g). A clear decrease of intensity is observed for the exposed sample, while the intensity remains constant within a 5% deviation after 1 week and within a 30% deviation after 1 month for the encapsulated one.

### Scalable Encapsulation *via* Semidry Transfer of Thin CVD-hBN

Due to the high reactivity of monolayer 1T′-MoTe_2_ in environmental conditions, a rapid and effective encapsulation method right after the growth is necessary to stabilize the material. In Figure 2a–d, the main steps of the encapsulation method proposed in this work are represented. First, commercially available hBN grown *via* CVD on copper is electrochemically delaminated from the growth substrate. To this end, a square of hBN larger than the target MoTe_2_/SiO_2_ sample is covered with a polymeric membrane of PMMA and PPC obtained *via* two sequential spin-coating and baking steps;^63^ then a PDMS frame a few millimeters thick is applied on top of the membrane (Figure 2a). The covered hBN is then delaminated *via* an electrochemical reaction in NaOH solution (Figure 2b). Once the delamination is completed, the detached membrane is rinsed in deionized (DI) water and dried. The PDMS frame on the top of the polymer/hBN membrane allows it to be safely manipulated in air and to be brought right next to the reactor in which the TMD has been grown. Finally, a fully dry, top-down encapsulation of the TMDs prevents any oxidation of the material (Figure 2c and d). Further details about this procedure can be found in the Methods section. Since a thick PDMS frame is employed, the lamination is performed in air without subjecting the 1T′-MoTe_2_ to water, which would be largely incompatible with the high reactivity of 1T′-MoTe_2_ in aqueous environments. In addition to the dry lamination step, the use of a thick PDMS frame allows safely bringing the hBN membrane right next to the reactor, so that the exposure to air of 1T′-MoTe_2_ is limited to the few seconds necessary to unload the sample, minimizing its degradation before encapsulation (further details are reported in the Methods section).

Since both the growth and the encapsulation methods reported in this work are fully scalable, a larger production of 1T′-MoTe_2_ is only limited by the section of the quartz tube loaded in the reactor. To demonstrate the full scalability of our approach, a 1.5 × 1.5 cm^2^ sample was grown and fully covered with hBN using the method reported in this work (Figure S5). After inspection of the encapsulated sample, the yield of coverage was found to be over 95% with a small percentage of tears and bubbles. We take advantage of these defects to study in parallel the properties of encapsulated and nonencapsulated 1T′-MoTe_2_ crystals on the same sample, as well as the tightness of the encapsulation next to the hBN tear or air bubble. To perform this parallel study, we use a MoTe_2_ sample obtained with a non-optimized growth (Figure S1a) to observe the different aging for different thicknesses. In Figure 2e the optical image of MoTe_2_ taken 1 week after the encapsulation on SiO_2_ is reported. A macroscopic air bubble present underneath the hBN layer is indicated in the figure by a red circle. Four different contrasts are visible on MoTe_2_ crystals, namely, white (W), dark blue (DB), blue (B), and pale blue (PB). In agreement with the literature,^30^ both the optical and the Raman analyses allow us to assign the white (W) and dark blue (DB) contrast to bulk MoTe_2_ and the B and PB contrasts to nonoxidized and oxidized monolayer MoTe_2_, respectively. It has been suggested in fact that the oxidation of MoTe_2_ takes place at the Te defects where environmental O_2_ is embedded, leaving Mo and Te in an oxidized state and the oxidized-MoTe_2_ structure intact.^28^ However, although no structural change is observed, the environmental oxidation of telluride crystals is usually accompanied by a fading of the blue contrast. While such fading is not observed for the bulk crystals exposed to air in panel e, it is evident for the monolayer regions within the red circle.

Moreover, for the crystals only partially exposed to air, a sharp increase of contrast at the edge of the bubble is observed, suggesting that 1T′-MoTe_2_ is tightly encapsulated and air infiltration is rather slow beneath hBN. Figure 2f and g report the optical image and a Raman intensity map of 1T′-MoTe_2_ encapsulated for 1 month in correspondence with an hBN tear. To confirm the local oxidation of MoTe_2_, the B_g_ peak at 163 cm^–1^ was chosen to monitor the degradation of the material (Figure 2g). As shown in Figure 1d, in fact, the B_g_ peak at 163 cm^–1^ is usually the most intense one, leading to the best signal-to-noise ratio and, in turn, minimizing any artifacts due to low Raman signals in oxidized areas (Figure S6). A sharp decrease of the Raman intensity of the B_g_ peak can be clearly observed in panel g in correspondence with the uncovered area, in perfect agreement with the tear visible in the optical image (black square in Figures 2f and S6). This result confirms not only that the adhesion level of hBN transferred on MoTe_2_ is extremely high but also that the air infiltration from air bubble or hBN tears is negligible, such that the oxidation of 1T′-MoTe_2_ remains localized to the exposed area, even after a month, corroborating the high quality of this encapsulation method. The hBN used in this work was also characterized after the same semidry transfer method on both bare SiO_2_ and MoTe_2_ samples by means of SEM, Raman, and AFM, and the results are summarized in Figure S7. Although a few tears and air bubbles are sometimes visible (Figure 2e), the yield of the transfer remains near the full coverage and is calculated to be over 95% on a 1.5 × 1.5 cm^2^ sample (Figure S5). The crystallinity of the hBN is also confirmed *via* Raman spectroscopy, and no appreciable change can be observed between hBN transferred on bare SiO_2_ or MoTe_2_ samples (Figure S7).

### Environmental Stability of Encapsulated 1T′-MoTe_2_

For further confirmation of the quality and stability of the encapsulation method reported here, the optical, structural, and electrical properties of encapsulated monolayer 1T′-MoTe_2_ have been monitored over several months and compared with monolayer 1T′-MoTe_2_ exposed to standard laboratory environmental conditions (22 °C and 30% humidity). In Figure 3 the optical images of exposed and encapsulated monolayer 1T′-MoTe_2_ are reported. As discussed above, the contrast of the MoTe_2_ can be used as a first tool to monitor the oxidation level of the MoTe_2_ single crystals. If monolayer 1T′-MoTe_2_ is exposed to air (Figure 3a–d), a first fading of the contrast is visible within 1 h from the growth, while complete oxidation takes place within 3 h, in good agreement with previous works.^30,61^ Again, the multilayer patches at the boundaries of different crystals do not show contrast variations, further confirming that the oxidation in few-layer 1T′-MoTe_2_ is much slower. If the 1T′-MoTe_2_ monolayer is encapsulated in hBN, instead, no contrast variation is observed in the first 3 h after encapsulation (Figure 3e–g), corroborating that, if the exposure of MoTe_2_ to air is prevented *via* hBN passivation, the degradation is largely reduced. Additional optical images of the encapsulated 1T′-MoTe_2_ after 1 week and 1 month from the encapsulation are illustrated in Figure S8. No significant oxidation is optically visible after 1 week from the encapsulation (Figure S8f). After 1 month, on the other hand, a dimmer contrast starts to be visible (Figure S8g). Since all the optical images are taken on the same sample for both the exposed and encapsulated MoTe_2_, this qualitative observation can be quantified by extrapolating the contrast intensity of 1T′-MoTe_2_ at different aging stages. Using the formula suggested by Naylor *etal*.,^61^ (*I*_MoTe_2__ – *I*_Sub_)/*I*_Sub_, where *I*_MoTe2_ is the contrast intensity of MoTe_2_ extrapolated in areas highlighted by the squares in Figure 3 a–g and *I*_sub_ is the contrast intensity of the substrate extrapolated from the surrounding area, the calculated values are plotted in Figure 3h for both the exposed and encapsulated sample at different aging times. As expected from previous observations, the contrast intensity of exposed 1T′-MoTe_2_ becomes 50% of the initial value after 1 h of exposure and decreases up to 90% after 3 h. The encapsulated sample, on the other hand, shows constant intensity with a decrease from 100% to 95% within the first week and to 69% after one month in air.

**Figure 4:**
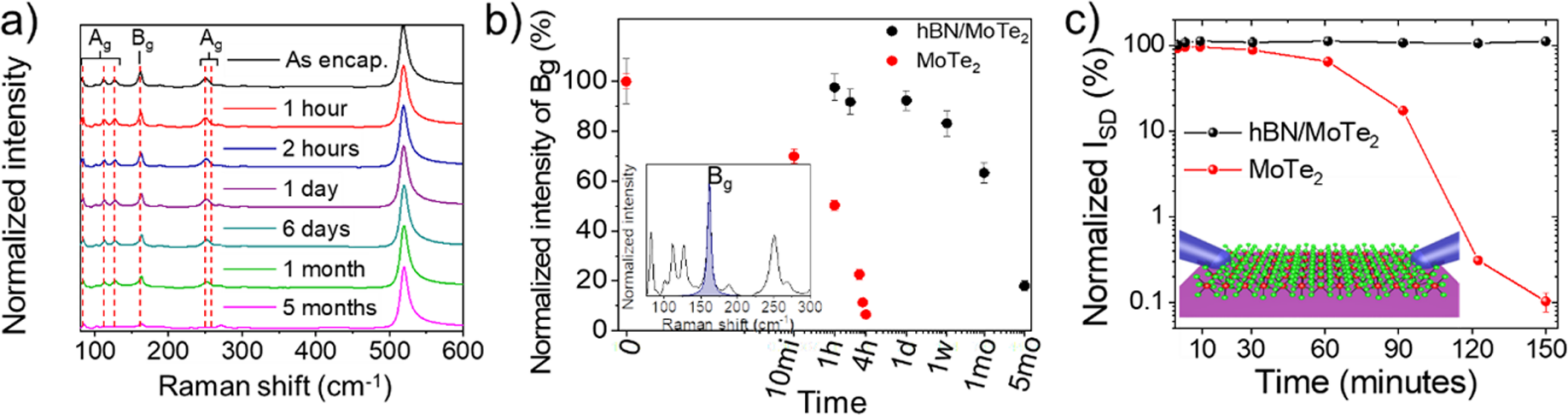
(a) Raman spectra of encapsulated 1T′-MoTe_2_ taken at increasing time after the encapsulation. (b) Intensity trend of the B_g_ peak at 163 cm^–1^ (inset) over aging time for the exposed and encapsulated 1T′-MoTe_2_ sample. The intensity is normalized with respect to the Si peak from the substrate, and the statistical values and errors are extrapolated from the single spectra of the Raman maps reported in Figures S9 and S10. (c) Variation over time of the current flowing through a single crystal exposed (red dots) and encapsulated (black dots) at *V*_SD_ = 0.1 V.

Further assessment of these observations has been carried out *via* micro-Raman mapping. In Figure 4a representative Raman spectra of hBN-encapsulated monolayer 1T′-MoTe_2_ at different stages of aging are reported. Since Raman maps are taken on the same 1T′-MoTe_2_ crystal, the intensity of a Raman peak, normalized with respect to the silicon (Si) peak intensity at 520 cm^–1^, can be employed to quantitatively monitor the degradation of 1T′-MoTe_2_. As reported above, the B_g_ peak at 163 cm^–1^ is chosen for its high signal-to-noise ratio, and in Figure 4b the intensity of the B_g_ peaks, normalized with respect to the intensity at time 0, is plotted over time. It is clear that if the sample is exposed to air (red dots in Figure 4b), the Raman intensity drastically reduces to about 50% in the first hour and reaches 0% within the first 4 h after growth, in good agreement with the optical results reported in Figure 3. The normalized intensity of the encapsulated sample (black dots in Figure 4b), instead, remains constant within the margin of error for the first week and decreases 30% after 1 month from the encapsulation, in perfect agreement with the optical results (red dots in Figure 3h). We also found that the normalized intensity of the encapsulated sample further decreases up to 80% after 5 months from the growth. This slow degradation of 1T′-MoTe_2_ when exposed to air for more than a month is probably due to small microbubbles and tears present in the hBN layer. This issue could be solved by using thicker (*e.g*., multilayer) hBN as encapsulant to prevent the formation of tears. Furthermore, thicker hBN could allow exploiting the self-cleaning mechanism in van der Waals heterostacks demonstrated previously for graphene–hBN heterostructures,^64,65^ which could lead to a bubble-free interface. The latter result could also be achieved by carrying out the encapsulation in an inert environment.^66^ The encapsulation reported in this work is in fact carried out in air, and therefore the membrane was not perfectly stretched before rapid lamination, leading to possible micro air bubbles trapped between hBN and MoTe_2_ layers. The use of a lamination setup in a controlled atmosphere is expected to minimize these air bubbles and, as a result, further increase the lifetime in air of the encapsulated material. It is, however, interesting to notice how the normalized intensity of the exposed sample after 3 h from growth is statistically comparable to the encapsulated sample after 5 months from encapsulation, corroborating that, even when thin hBN is used, the lifetime of the 1T′-MoTe_2_ is significantly increased by this encapsulation method. The complete Raman normalized intensity maps of the encapsulated and exposed samples are reported in Figures S9 and S10, respectively.

The aging of the 1T′-MoTe_2_ monolayer has also been evaluated by monitoring the current flowing through the crystals at a fixed voltage over time. In order to compare the electrical behavior of exposed and encapsulated 1T′-MoTe_2_ monolayer, the characterization of the electrical properties has been performed for both samples using a custom-made probe station. In this case a pair of probe station needles are put in direct contact with the crystal, through micrometric manipulators. This approach was necessary because of the limited lifetime of exposed monolayer 1T′-MoTe_2_, which does not allow performing the fabrication of metallic contacts using electron or optical lithography and metal evaporation without largely reducing the quality of the material in comparison to stable encapsulated 1T′-MoTe_2_. This approach indeed allows us to perform electrical characterization on the exposed 1T′-MoTe_2_ only a few minutes after the growth, minimizing the effect of oxidation prior to the measurement. The natural elongated shape of the single-crystal 1T′-MoTe_2_ is suitable for a simple two-terminal measurement of the resistivity through a naturally defined channel. In Figure 4c we report the time evolution of the current flowing in both exposed and encapsulated 1T′-MoTe_2_ single crystals. The measurement was performed applying a constant bias *V*_SD_ = 0.1 V to the needles directly in contact with the sample, while the flowing current has been continuously measured for about 3 h. For both samples, the initial current *I*_0_ is in the range of hundreds of nA (see Supporting Information for further details). Other monolayer 1T′-MoTe_2_ crystals for both samples have also been measured for shorter time, showing initial currents of the same order of magnitude, with variations that can be attributed to the different distances between the needles (25 and 35 μm for exposed and encapsulated sample, respectively), which cannot be controlled below a few micrometers (system limits) (Figure S11a and b). For the sake of comparison, the reported acquisitions of flowing current in Figure 4c have been chosen to match the aging times used for the other characterization techniques reported above. These values were obtained by averaging over 50 acquisitions, in a time of 60 s, to reduce the variation in the recorded signal arising from mechanical oscillations in the acquisition setup, as shown in Figure S11c. The degradation of the exposed sample, compared with the encapsulated one, is clearly shown by the decrease of the current reported in Figure 4c. The measurement for the exposed 1T′-MoTe_2_, in red in Figure 4c, started right after growth and exposure of the sample to air; while for the first minutes the variation remains within the error (Figures 4c and S11c), after 30 min the reduction in the flowing current is no longer compatible with the signal fluctuation. Then, the conductivity drops down in an exponential manner for the following 2 h. After around 2.5 h from the growth, the current flowing through the crystal is below 100 pA, which is the measurement limit of the setup. The conductivity of the encapsulated sample, black dots in Figure 4c, instead, remains comparable to the initial values in the same time interval. Moreover, the encapsulated sample showed no relevant changes in the electrical behavior several days after the encapsulation.

### Electrical and Low-Temperature Properties of Encapsulated 1T′-MoTe_2_

We further carried out electrical characterization of the material on fully fabricated encapsulated samples by studying the dependence of the conductivity on field-effect doping and temperature. To avoid exposing the encapsulated material to air, we directly fabricated the metal contacts on top of hBN/1T′-MoTe_2_, so that the device operates through a tunneling junction. Reactive ion etching was only performed far from the contacted single crystal (Figure S12a) to clean the substrate, to promote metal adhesion, and to avoid possible short-circuiting of the contact, all factors that could affect the measured conductivity. The natural elongated shape of the 1T′-MoTe_2_ crystal, indeed, allows having a naturally defined channel. Raman mapping of the encapsulated 1T′-MoTe_2_ monolayer after full fabrication of transfer length measurement (TLM) contacts as well as more complex devices (*i.e*., Hall bars) confirms that, if encapsulated, 1T′-MoTe_2_ can undergo all the required fabrication steps with no significant degradation of the material quality (Figure S12).

**Figure 5:**
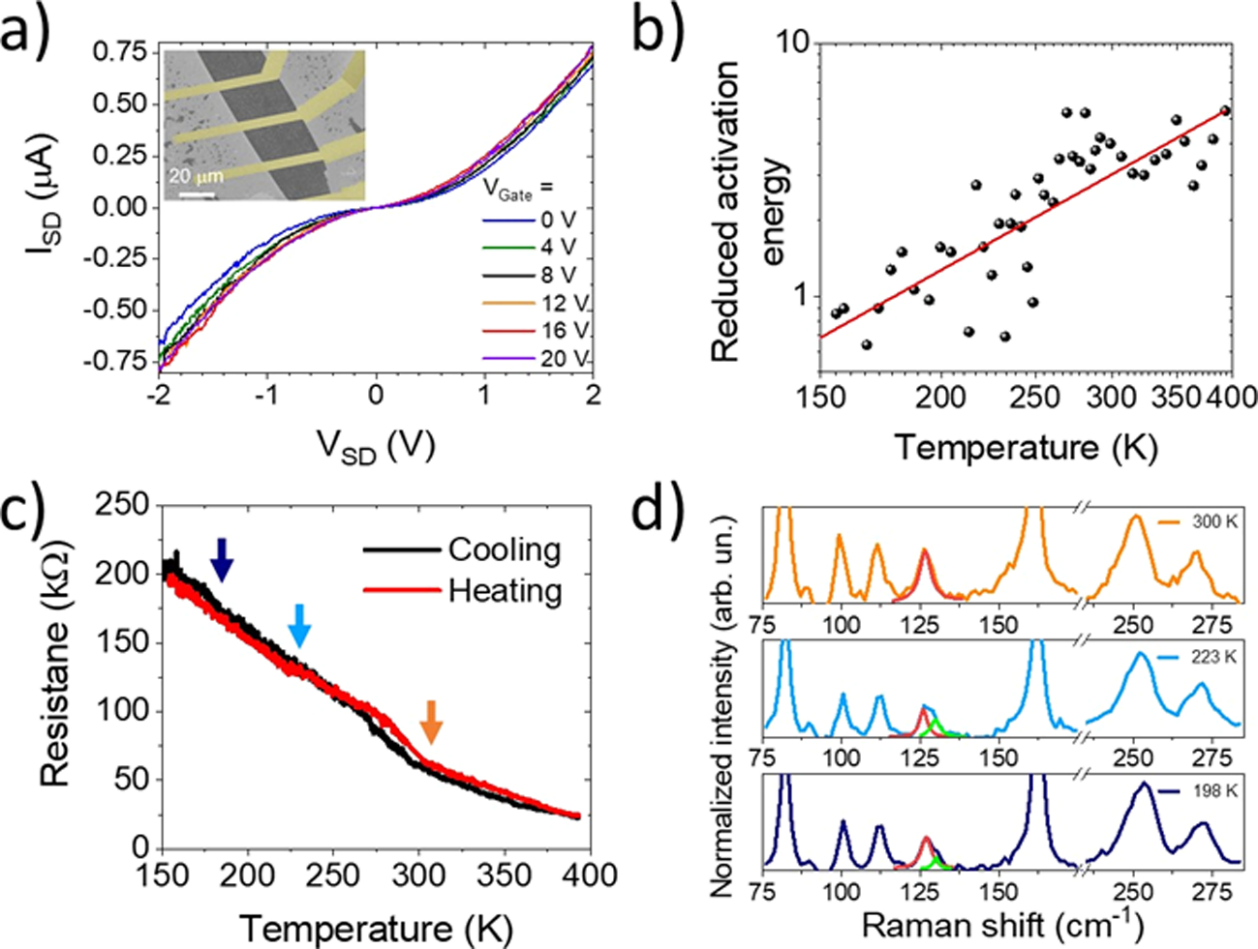
(a) *IV* curves of encapsulated 1T′-MoTe_2_ after device fabrication collected at different applied back-gate voltages, compatible with the metallic nature of monolayer 1T′- MoTe_2_.^72^ The nonlinearity of the *IV* curves is due to the insulating hBN layer between MoTe_2_ and the metal contacts. Inset: false-color SEM image of the device with a form factor of ∼1. (b) Reduced activation energy dependence on the temperature in the range 150–375 K, plotted in log–log scale. The positive slope of *W*(*T*) in this range confirms the metallic nature of the material.^73^ (c) Temperature-dependent resistance of a monolayer 1T′-MoTe_2_ single crystal, measured at an applied constant bias of 1 V (chosen to reduce the tunneling barrier effect). Hysteresis between cooling and heating is visible around 250–300 K, which suggests the phase transition from 1T′ to T_d_ MoTe_2_.^76^ (d) Raman spectra acquired at 300, 223, and 198 K (highlighted by the orange, light blue, and dark blue arrows in panel c, respectively). A splitting of the peak at 129 cm^–1^ is visible, a signature of MoTe_2_ phase transition.^77^ The peak at 270 cm^–1^ confirms the monolayer nature of the crystal under study.^14^ All the temperature-dependent measurements are performed at ambient pressure, in a dry nitrogen environment.

In Figure 5a the *IV* curve on one representative fabricated device on 1T′-MoTe_2_ one month after the encapsulation is shown. As expected, the presence of the hBN layer between the metal contacts and the conductive MoTe_2_ induces a tunnel barrier,^67^ easily detectable in the nonlinear behavior of the *IV* characteristics.^68,69^ A more detailed characterization of the tunneling junction at the interface is reported in Figure S13. From resistance measurements the thickness of the hBN is confirmed to be about 3 or 4 layers, in agreement with AFM characterization (Figure S13a) and previous works employing similar commercially available hBN.^70,71^ No significant gate dependence is visible in the *IV* curves shown in Figure 5a when different back-gates are applied, which was also confirmed in the four-probe transfer curve reported in Figure S12c, suggesting that the MoTe_2_ under study has semimetallic behavior, in agreement with previous studies.^14,41,72^

The conductivity of the material and its dependence on temperature has further been studied as a function of temperature, from 150 to 380 K, at ambient pressure, in a dry nitrogen atmosphere (more details in the Methods section). In Figure 5b the reduced activation energy, calculated as , *versus* temperature is reported in log–log scale.^73,74^ The slope of the reduced activation energy (*W*(*T*) as a function of *T*) is positive and higher than 1 in a log–log plot, a further confirmation of the semimetallic behavior of monolayer 1T′-MoTe_2_.^41,74,75^ In Figure 5c the dependence of the resistance on the temperature is plotted; in order to limit the tunneling barrier effect, we perform the measurement in a high constant bias regime (1 V). The negative slope in panel c is likely due to the contribution of the metal/hBN/1T′-MoTe_2_ multiple interfaces (Figure S12f) and has been reported also for other 1T′-MoTe_2_ devices.^41^ A step is also visible in the 250–300 K range, due to a change of the resistance dependence upon temperature. This step is observed in both the heating (red line) and cooling (black line) ramps, resulting in a hysteretic behavior. This hysteretic behavior has already been reported^76−80^ and is a signature of a phase transition between the room-temperature metastable monoclinic phase (1T′) and the orthorhombic (T_d_) phase, which is stable at lower temperature.^81,82^ For further investigation of this signature, Raman spectroscopy was also carried out at different temperatures. It has in fact been reported that due to the change of symmetry from 1T′ to T_d_ structure, this phase transition is also accompanied by a splitting of the A_g_ peak at 129 cm^–1^ (also known as the P_6_ peak), in P_6A_ and P_6B_ peaks.^77^ Similar to the Raman spectra reported for bulk 1T′-MoTe_2_,^76^ in Figure 5c this splitting is visible (red and green fitting curves), suggesting that at temperatures lower than 223 K the phase is T_d_. To the best of our knowledge, such a phase transition was not yet observed on pristine monolayer 1T′-MoTe_2_. Paul *etal*.^77^ recently studied the dependence of this phase transition on the thickness, but concluded that it is hindered by the oxidation of the monolayer if exposed to air, unless the material was first chemically treated.^77^ The results observed here are a further confirmation that the quality of the grown material, as well as the stabilization method proposed in our work, allows one to investigate fundamental properties of this enticing material that were before out of reach. Further investigation of encapsulated MoTe_2_ in the limit of monolayer and of its properties at low temperature will be carried out in the future. Furthermore, as shown by the Raman map reported in Figure S12e, the quality of encapsulated CVD-grown monolayer 1T′-MoTe_2_ is preserved even after multiple heating and cooling cycles; this further broadens the characterization possibilities and applicative prospects of this material.

## Conclusions

In summary, here we described a fully scalable growth and encapsulation method that will help to carry out fundamental studies and increase a technologically oriented adoption of monolayer 1T′-MoTe_2_. Using an optimized LqP-CVD process, together with an appropriate substrate preparation, large-area monolayer 1T′-MoTe_2_ crystals with a lateral size of several hundreds of micrometers can be homogeneously obtained on SiO_2_ substrates of chosen dimensions. Such crystal size allows for the simultaneous fabrication of a large number of quantum or spintronic devices^44,83^ and is compatible with the typical dimensions of 2D-based photonic devices.^84,85^ Further scalability of this approach could be obtained by implementing deterministic crystal nucleation on the substrate.^86−88^

The dry lamination step demonstrated to encapsulate MoTe_2_ with delaminated CVD-grown hBN allows avoiding contact with liquids, which would induce a rapid deterioration of the as-grown sample. Because of the use of the thick PDMS frame during the transfer, the lamination steps can be carried out next to the reactor, so that the exposure to air of the as-grown 1T′-MoTe_2_ is minimized to the few seconds necessary to unload the sample. In this way, we have demonstrated by means of microscopic and spectroscopic analyses that the lifetime of the material is increased from a few minutes to more than a month. Several 1T′-MoTe_2_ samples have been encapsulated using this method, and their stability has been monitored over a few months. Although a few holes or air bubbles are sometimes visible in the hBN layer, the yield of encapsulation, easily assessable *via* the different contrast of oxidized and nonoxidized monolayer 1T′-MoTe_2_, remains more than 95% over samples as big as 1.5 × 1.5 cm^2^, suggesting that further scalability is mainly limited by the size of the reactor. Furthermore, the high level of tightness of the encapsulation has been confirmed by means of Raman mapping, and the air infiltration from bubbles remains under control for 1 month. Due to its flexibility and scalability, we suggest that this encapsulation method could be straightforwardly employed as a reliable route for stabilization of other reactive 2D materials.

The presented encapsulated monolayer 1T′-MoTe_2_ is compatible with the time scales of most characterization techniques and device fabrication steps, without large degradation of the starting material. In fact, electrical characterization on fully fabricated devices indicates metallic behavior, together with signatures of low-temperature 1T′–T_d_ phase transition. This suggests that the encapsulation *via* hBN reported here does not influence the exotic properties of the material, while largely improving its environmental stability, and can be employed to investigate the electrical and structural properties of 1T'-MoTe_2_ under different conditions. It is also worth noting that all the results reported here are obtained on samples encapsulated and stored in standard laboratory environmental conditions (22 °C and 30% humidity). Therefore, improved results are expected if the same method is used with the aid of a glovebox or controlled atmosphere and if the encapsulated sample is stored in a vacuum, so that the degradation of the material before and after encapsulation can be further minimized.

## Methods

### Growth of 1T′-MoTe_2_ Using Liquid-Precursor Chemical Vapor Deposition

The 1T′-MoTe_2_ samples were grown *via* liquid precursor CVD. In this method, the molybdenum precursor is obtained from an aqueous solution directly spun on the growth substrate. First three mother solutions were prepared, namely, solutions A, B, and C. Solution A was obtained by dissolving 0.15 g of AHM (Sigma-Aldrich) in 40 mL of DI water, solution B was obtained by dissolving 0.1 g of NaOH (Sigma-Aldrich) in 40 mL of DI water, while solution C consists of OptiPrep (Sigma-Aldrich) used as purchased. The growth solution was obtained by mixing the mother solutions with the ratio A:B:C equal to 1:1:0.5. The growth solution was then spun on a clean SiO_2_/Si substrate at 2900 rpm for 1 min. Before spin-coating, all SiO_2_ substrates were cleaned *via* standard sonicated cleaning in acetone and isopropanol for 5 min; moreover some SiO_2_ substrates were treated right before the spinning step using an additional step in oxygen plasma (power 100 W, process pressure 80 mTorr) for 5 min to enhance the hydrophilicity of the SiO_2_ surface. The spin-coated substrate and the metallic tellurium were then loaded in a Lenton hot-wall horizontal CVD reactor as shown in Figure 1a. The growth was carried out at near-atmospheric pressure and 730 °C for 15 min, under constant flow of Ar/H_2_ (H_2_ 3%) gas at 100 sccm. After the growth, the reactor was allowed to cool naturally and under constant flux of Ar (Figure 1b).

### Semidry Encapsulation Method

Semidry encapsulation of as-grown 1T′-MoTe_2_ was obtained *via* delamination of CVD hBN grown on copper foil adapted from refs (86) and (89). Nominally monolayer hBN (15 × 15 cm^2^) was purchased from Graphene Supermarket and directly used as received. The hBN was cut in squares of dimensions larger than the target SiO_2_ sample (*e.g*., 2 × 2 cm^2^ hBN for 1.5 × 1.5 cm^2^ MoTe_2_/SiO_2_ samples). Then, it was covered with a double-layer polymeric membrane obtained by two sequential steps of spinning and baking at 90 °C of PMMA AR-P 679.02 (Allresist) and 15% PPC in anisole (Sigma-Aldrich). A PDMS frame a few millimeters thick was applied on the top of the sample. The sample was then immersed in a NaOH solution (1 M), where an electrochemical reaction takes place: here the hBN/copper foil stack is the working electrode and a platinum foil is used as counter electrode and a constant −2.5 V voltage is applied until complete delamination. An intermediate cleaning step in water for 5 min was used to remove the NaOH; then the detached membrane was directly laminated on top of the as-grown sample and heated at 90 °C. Finally, a standard double cleaning in acetone and isopropanol was used to remove the supporting polymer (Figure 2a–d).

### Characterization Techniques

The optical images of the exposed and encapsulated 1T′-MoTe_2_ samples were obtained using either a Nikon or a vibrationally isolated Leica microscope with 10×, 20×, or 50× lenses. For the optical images at different aging steps, the exposure was set to 70 ms and the contrast was optimized using the hBN/SiO_2_ substrate as reference. Raman characterization was carried out with a Ranishaw InVia system. The laser wavelength used was 532 nm with a laser spot of ∼1 μm and a 100× lens. The Raman maps were obtained with a pixel step of 1 μm, laser power of 5 mW, and exposure time of 1 s. The AFM data were acquired with a Bruker Dimension Icon microscope in PeakForce quantitative nanomechanical (QNM) mode.

Electrical characterization of the 1T′-MoTe_2_ was performed using a custom-made probe station on an actively isolated optical table. The metal probe needles (MPI-Corporation 7 μm tungsten needles) were placed directly in contact with the crystals using micrometric positioners (MPI MP40 MicroPositioner) with a screw step of 300 μm. To perform the measurements, the metallic needles of the probe station were carefully approached to a visible isolated crystal, until a stable current was observed; from that moment the measurement was continuously performed for 3 h. The optical selection of the 1T′-MoTe_2_ crystal and the control of the movement of the needles were performed using an optical microscope orthogonal to the sample, with 10× magnification. Particular care was taken to avoid moving the conductive needles or the sample: to minimize the mechanical oscillations, the measurement setup was mounted on a pneumatic isolated optical table (with Newport I-2000 pneumatic isolation legs), with all the electrical cables secured in a fixed position. The contact area of the needles is visible in the SEM image in Figure S11a and b. To optimize the time of measurements, particularly in the case of exposed MoTe_2_, the measurements were performed at the same time on two crystals close-by, using a pair of Keithley 2450 source-meters. To perform the measurement of the conductivity directly on the crystals, we proceeded as follows: we moved the needles close to each other (in the range of a few hundred micrometers) and moved down on the SiO_2_ bare substrate, with the voltage generators set at zero. Here the needles are lifted by a known amount (half-twist of the micrometric positioner) and then moved over the chosen crystal. Then the source–drain voltage was set to the target value (0.1 V) and the needles were carefully moved down to the same height as before or until a signal was detected on the source-meter; this protocol assured, for the encapsulated MoTe_2_, that the needles drilled the hBN insulating layer as shown in Figure S11b.

### Fabrication Methods

Encapsulated monolayer 1T′-MoTe_2_ was transferred on different substrates for both AFM and electrical characterization *via* HF transfer. The sample was first covered with a polymeric membrane obtained by spinning Allresist 679.02 PMMA and baking at 90 °C for 2 min. The sample was then left floating for a few seconds in concentrated HF until complete detachment. Once detached, the floating polymeric membrane is fished from the HF solution and rapidly rinsed in water for a few seconds. Finally, the membrane is fished again using the target substrate, *i.e*., clean SiO_2_/Si substrate, and baked again at 90 °C for 1 min. The polymer was then removed *via* standard cleaning in acetone, leaving clean hBN/MoTe_2_ on the target substrate.

The full fabrication process was carried out only on encapsulated MoTe_2_, since it is stable enough to allow the fabrication of electrical devices *via* standard electron or optical beam lithography and metal evaporation. The size of the single crystals allows the realization of devices with up to eight terminals, on a single crystal, with resolution compatible with optical lithography.

A two-step lithography process has been used to realize both TLM and eight-terminal devices (as shown in Figure S12). First EBL or optical lithography was used to define the device area and reactive ion etching (CF_4_/O_2_ 20/2 sccm) to remove the additional crystals around the chosen one, in order to avoid multiple conduction channels and to allow a better adhesion for the contact metals. A second lithographic step was employed to realize metal pads and metal contacts on the 1T′-MoTe_2_ crystal, *via* thermal evaporation of 50 nm of gold and 5 nm of chromium as adhesion layer. Due to the naturally elongated nature of the MoTe_2_ crystals, no etching step has been used to define the channel shape.

This fabrication procedure was performed both on the growth substrate and on the transferred sample.

### Low-Temperature Characterization

Temperature-dependent electrical characterization was performed in a Linkam heat/cooling stage (HFS600-P), which allows cooling the sample holder to about 100 K *via* liquid nitrogen flux or heating to about 700 K *via* a resistive thermal element, while simultaneously characterizing the electrical properties of the material using tungsten tips covered in gold. The Raman investigation at low temperature was carried out in the same chamber on the same sample and with a 50× long-focal length lens. In both characterization procedures, the presence of oxygen and water contamination, which might lead to ice or dew in the chamber at a temperature near the temperature of interest for the transition phase, was prevented *via* a purging procedure that replaced the ambient atmosphere with dry nitrogen evaporated from the cooling loop. Electrical characterization was carried out using a DC source-meter (Keithley 2450), and two-probe measurements were performed due to substrate space limitations in the chamber.
