# Supplementary material for: Synthesis of Large-Scale Monolayer 1T′-MoTe2 and Its Stabilization via Scalable hBN Encapsulation
Source: ACS Nano. 2021 Feb 19;15(3):4213–25. doi: 10.1021/acsnano.0c05936 (PMC8023802; doi:10.1021/acsnano.0c05936)
Supplement: Supplementary file 1 — nn0c05936_si_001.pdf [file nn0c05936_si_001.pdf]

Synthesis of Large-Scale Monolayer 1T'-$MoTe_2$ and Its Stabilization *via* Scalable hBN Encapsulation

Simona Pace [1, 2, *], *Leonardo Martini* [1], *Domenica Convertino* [1,2], *Dong Hoon Keum* [1, 2], *Stiven Forti* [1], *Sergio Pezzini* [1, 2, 3], *Filippo Fabbri* [1, 2, 3], *Vaidotas Mišeikis* [1, 2,] *Camilla Coletti* [1, 2, *]

[1] Center for Nanotechnology Innovation @NEST - Istituto Italiano di Tecnologia, Piazza San Silvestro 12, 56127 Pisa, Italy

[2] Graphene Labs, Istituto Italiano di Tecnologia, Via Morego 30, 16163 Genova Italy

[3] Laboratorio NEST, Istituto Nanoscienze-CNR, Piazza San Silvestro 12, 56127 Pisa, Italy

[*] Corresponding authors: simona.pace@iit.it, camilla.coletti@iit.it

SUPPORTING INFORMATION

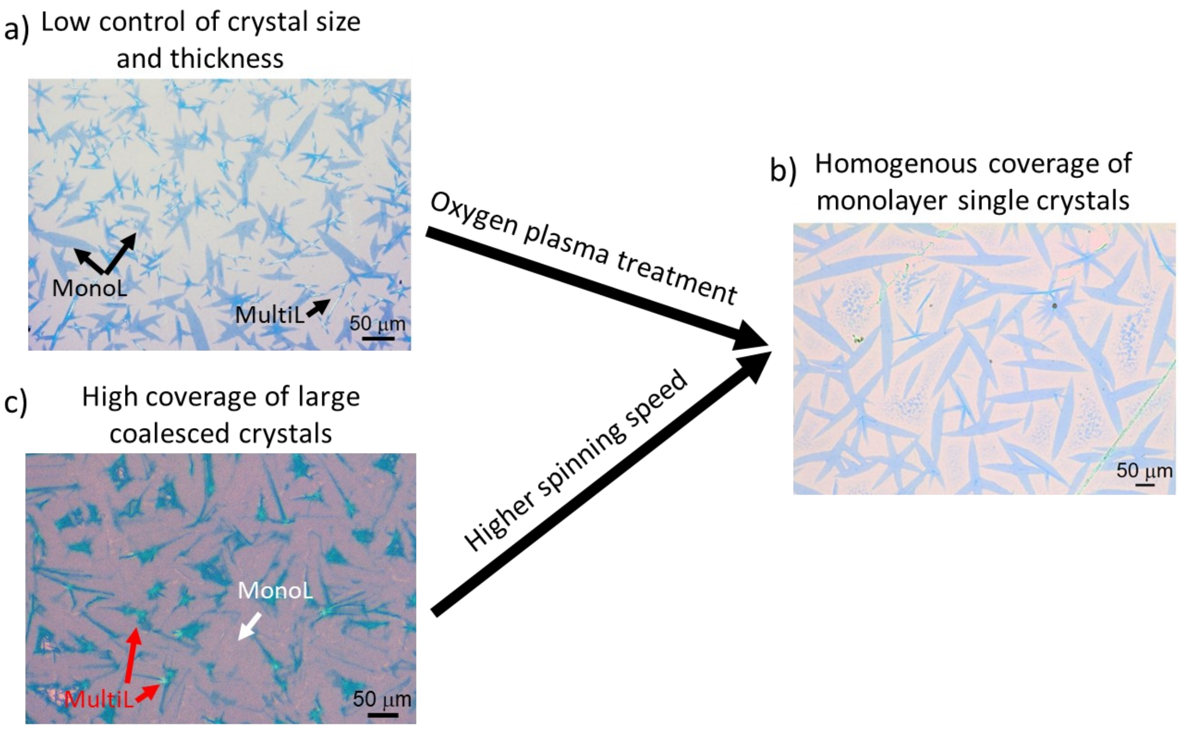

Figure S1 Optimization of the pre-treatment of the substrate. a) Optical images of 1T'-$MoTe_2$ grown on (a) not-treated and (b) pre-treated substrate using oxygen plasma in addition to standard cleaning steps. The use of oxygen plasma increases the hydrophilicity of SiO_2 surface, improving the homogenous distribution of the growth solution during the spinning step. As a result of this pre-treatment, larger monolayer 1T'-$MoTe_2$ crystals are obtained and less or no bulk crystal are observed (panel b). c) Optical image of 1T'-$MoTe_2$ grown on a pre-treated substrate using a lower spinning speed. If the spinning speed is lowered from 2900 rpm (panel b) down to 1900 rpm (panel c), the amount of solution spun on the substrate is increased and the coverage can be progressively increased up to full coverage. Although few multilayer patches are visible (red arrows) the coverage of monolayer is still about 80% of the area (estimated using a particle analysis at a set threshold).

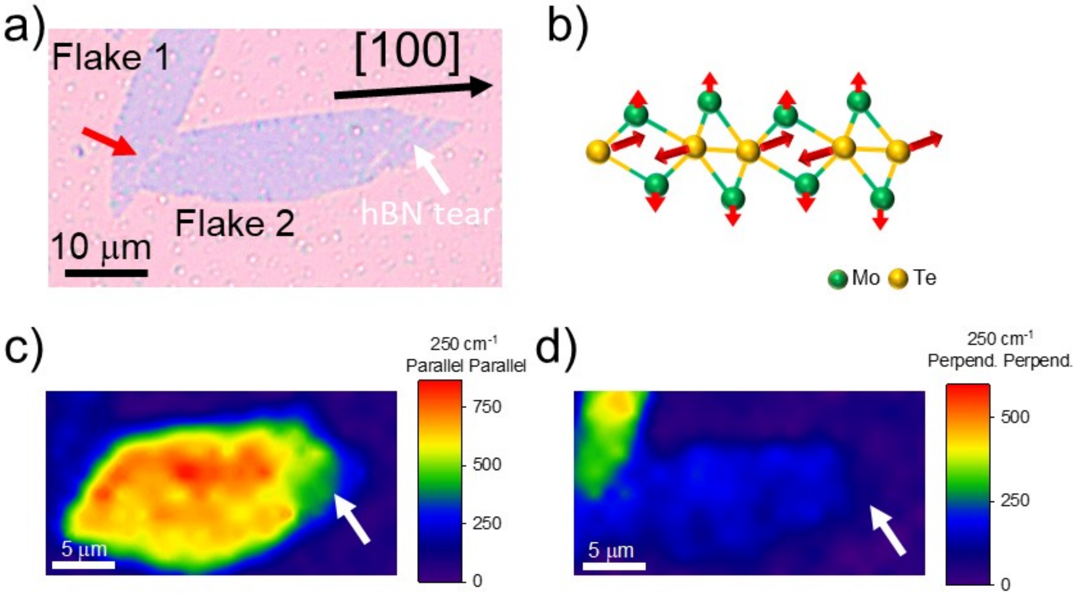

Figure S2 Polarized Raman of encapsulated 1T'-MoTe$_2$. a) Optical image of two coalesced 1T'-MoTe$_2$ crystals (flake 1 and 2). In 1T'-MoTe$_2$ the grain boundaries are extremely reactive and quickly oxidize before the encapsulation (red arrow); as such optical images of these defects can be used as rapid tool for a first evaluation of the crystallinity level of 1T'-MoTe$_2$. Using this first evaluation tool, in panel a, only one grain boundary is visible, suggesting that each elongated flake is single-crystal.

Due to the in-plane anisotropy of the 1T'-MoTe$_2$ crystal structure, the intensity of the Raman peaks collected with polarized excitation is strictly dependent on the crystal orientation. Therefore, polarized Raman is used to further assess the single-crystal nature of the 1T'-MoTe$_2$ flakes studied in this work. Since it has been reported that the intensity of the in-plane A_g 250 cm^{-1} mode (schematic representation in panel b) is highly sensible to the orientation of both the laser and the analyzer, [1] this Raman mode was chosen for this evaluation. In panels c and d the intensity maps of the 250 cm^{-1} mode, are reported for flakes 1 and 2. The laser and analyzer are set parallel (panel c) and perpendicular (panel d) to the [100] crystal direction of flake 1. From panel c it is clear that the 250 cm^{-1} intensity is largely enhanced if both laser and analyzer are parallel to the [100] direction of 1T'-MoTe$_2$ and quickly decreases if 1T'-MoTe$_2$

is misoriented (flake 2). Instead, the 250 cm^{-1} intensity is quenched if both laser and analyzer are perpendicular to the [100] direction (panel d). Moreover, no clear intensity step is visible within flake 1 and flake 2, while a large intensity difference is visible between the two flakes in both maps, confirming that each elongated flake is single-crystal 1T'-$MoTe_2$. In both panels c and d, a slight decrease of intensity is visible on the tip of flake 1 (white arrows), and attributed to a partial oxidation of the flake caused by a hBN tear visible in the optical image (also highlighted by the white arrow in panel a).

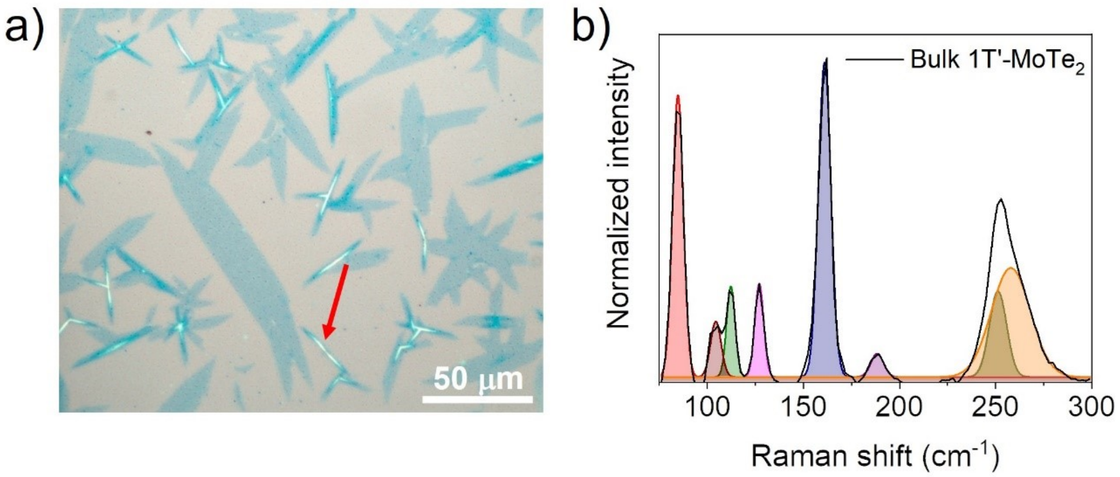

Figure S3 Characterization of bulk 1T'-MoTe₂. a) Optical image of 1T'-$MoTe_2$ grown using liquid precursor chemical vapor deposition (LqP CVD) on SiO_2 substrate not-treated with oxygen plasma prior to the spinning. Both monolayer (blue contrast) and bulk (white contrast) crystals are visible. b) Representative Raman spectrum taken on the 1T'-$MoTe_2$ crystal highlighted by the red arrow in panel a. The fitting of the different 1T'-$MoTe_2$ vibrational modes are highlighted in different colors. If compared with the spectrum in Figure 1d, a clear redshift of the A_g peak from 269 cm^{-1} (monolayer) to 260 cm^{-1} (bulk) is visible. [2,3]

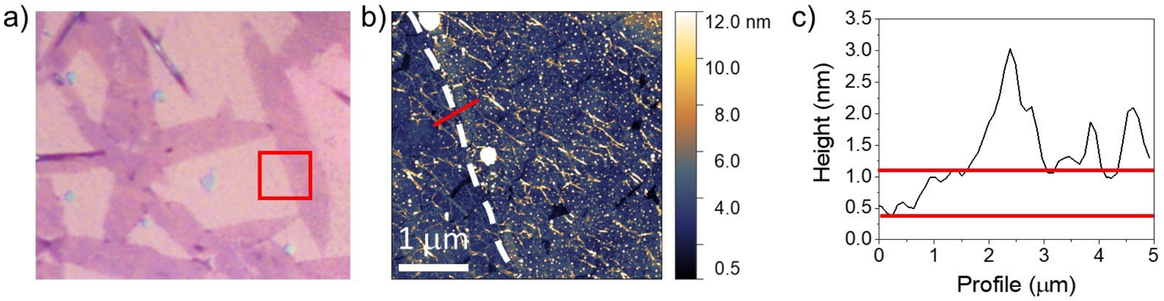

Figure S4 AFM analysis of encapsulated 1T'-$MoTe_2$. a) Optical image of transferred 1T'-$MoTe_2$ on clean SiO_2 substrate. b) AFM micrograph of hBN-encapsulated monolayer 1T'-$MoTe_2$, after transfer on SiO_2, performed in the area highlighted by the red square in panel a. A clear step is visible between outside (left side) and inside (right side) the $MoTe_2$ crystal. Some levels of roughness are observed both inside and outside 1T'-$MoTe_2$ single-crystal, probably due to PMMA residuals deposited during the encapsulation, as well as intrinsic roughness of the hBN used (see Figure S7 for further details). On the studied area, small particles (white in contrast) decorating the edge of the flake are also visible, which are expected to be growth by-products, as already observed on other TMDs grown using similar techniques. [4] c) Line profile of hBN/$MoTe_2$ extracted from panel b (red line). A clear step of about 8.2 ±0.4 Å is visible and compatible with the monolayer thickness of 1T'-$MoTe_2$ within the error of the measurement, [3] corroborating the Raman results reported in the main text. The hBN encapsulant is continuous over the entire examined area and does not contribute to the measured step height.

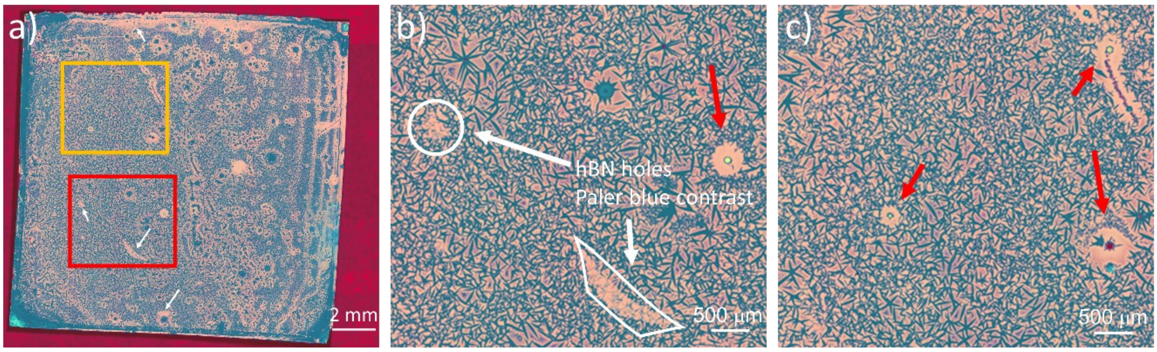

Figure S5 Homogenous growth of 1T'-$MoTe_2$ over large area. a) Large area optical image taken on a 1.5 x 1.5 cm^2 sample of 1T'-$MoTe_2$ encapsulated with hBN using the method reported in this work. In order to have full coverage, an hBN membrane bigger than the sample was used so that the crystals on the edge were also encapsulated. Areas with hBN tears and holes are characterized by the presence of both a paler blue contrast (oxidized $MoTe_2$) and a brighter pink contrast (exposed SiO_2). b) Zoom-in of the area highlighted in panel a by the red square where defective hBN was found. The areas indicated by the white arrows show dimmer contrast for 1T'-$MoTe_2$ oxidized crystals (pale blue color) and brighter contrast for the exposed SiO_2 (pink color). c) Zoom-in of the area highlighted in panel a by the yellow square. Continuous hBN encapsulation is found on this area where no tears or bubble are observed. Bright pink contrast in panels b and c is observed in areas where growth did not take place (bulk agglomerates are visible in the centre of the bright pink spots (indicated by red arrows). Accurate analysis of the sample indicate that tears and bubbles are present on less than 5% of the 1.5 x 1.5 cm^2 sample. This value is in agreement with the nominal hBN coverage provided by the seller. [5]

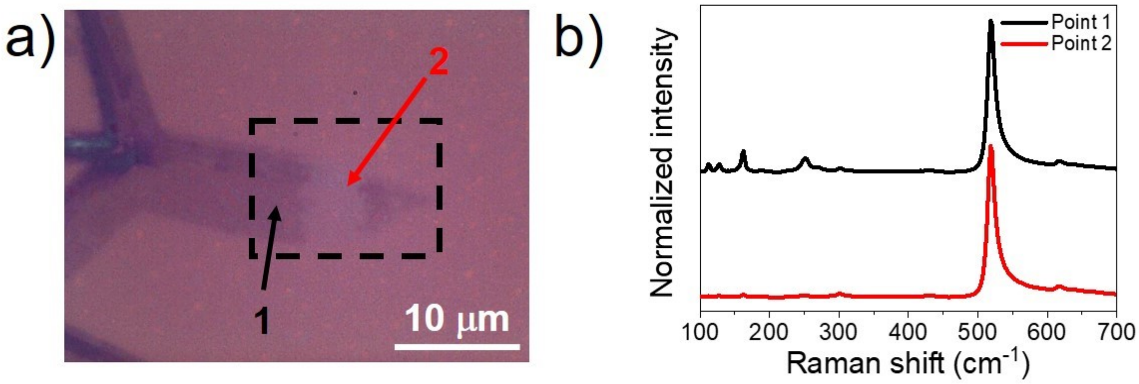

Figure S6 Characterization of the tightness of the encapsulation. a) Optical image of single crystal 1T'-$MoTe_2$ after 1 month from the encapsulation in hBN. Where a hBN tear is visible, a clear contrast difference is observed within the crystal, due to the oxidation of $MoTe_2$ exposed to air. b) Representative single Raman spectra taken at Point 1 and 2 right next and within the tear, as highlighted in panel a.

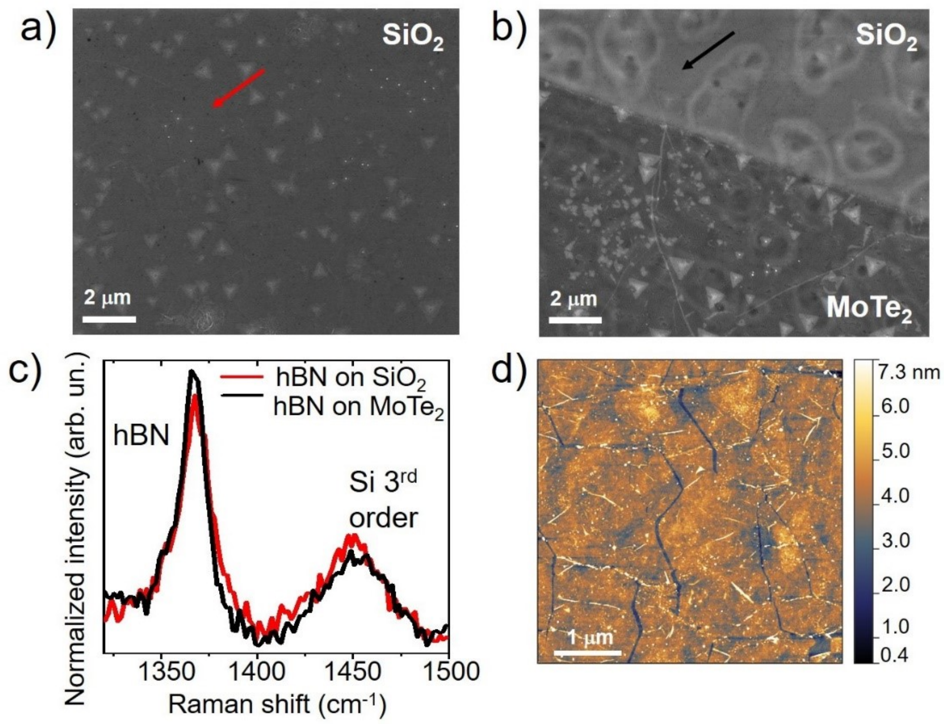

Figure S7 Characterization of hBN. a – b) SEM images of CVD hBN purchased from *"Graphene-Supermarket"* and transferred on bare SiO_2/Si substrate (a) and 1T'-$MoTe_2$ (b) using the semi-dry encapsulation method proposed in this work. Clear bulk triangles are visible on both sample, as expected from the specifics reported by the seller. c) Raman single spectra of hBN after transfer on bare SiO_2/Si substrate (red spectrum) and $MoTe_2$ sample (black spectrum) taken in the area highlighted by the arrows in panel a and b, respectively. The intensities of these spectra have been normalized with respect to the third order of Si peak at 1450 cm^{-1}. hBN peaks on bare SiO_2/Si and $MoTe_2$ show a full width half maximum (FWHM) of 13.9 ± 0.9 and 15.9 ± 0.6 cm^{-1}, respectively. d) AFM image of hBN transferred on bare SiO_2/Si substrate. White wrinkles are visible (also in panel b) as well as small cracks. Those might be due to the need of quickly encapsulating the $MoTe_2$ sample using the polymeric membrane in environmental conditions before oxidation of $MoTe_2$. Such that, the lamination must be done without slowly approaching the surface and before the membrane is fully stretched. This can be easily overcome by carrying out the lamination process in controlled atmosphere.

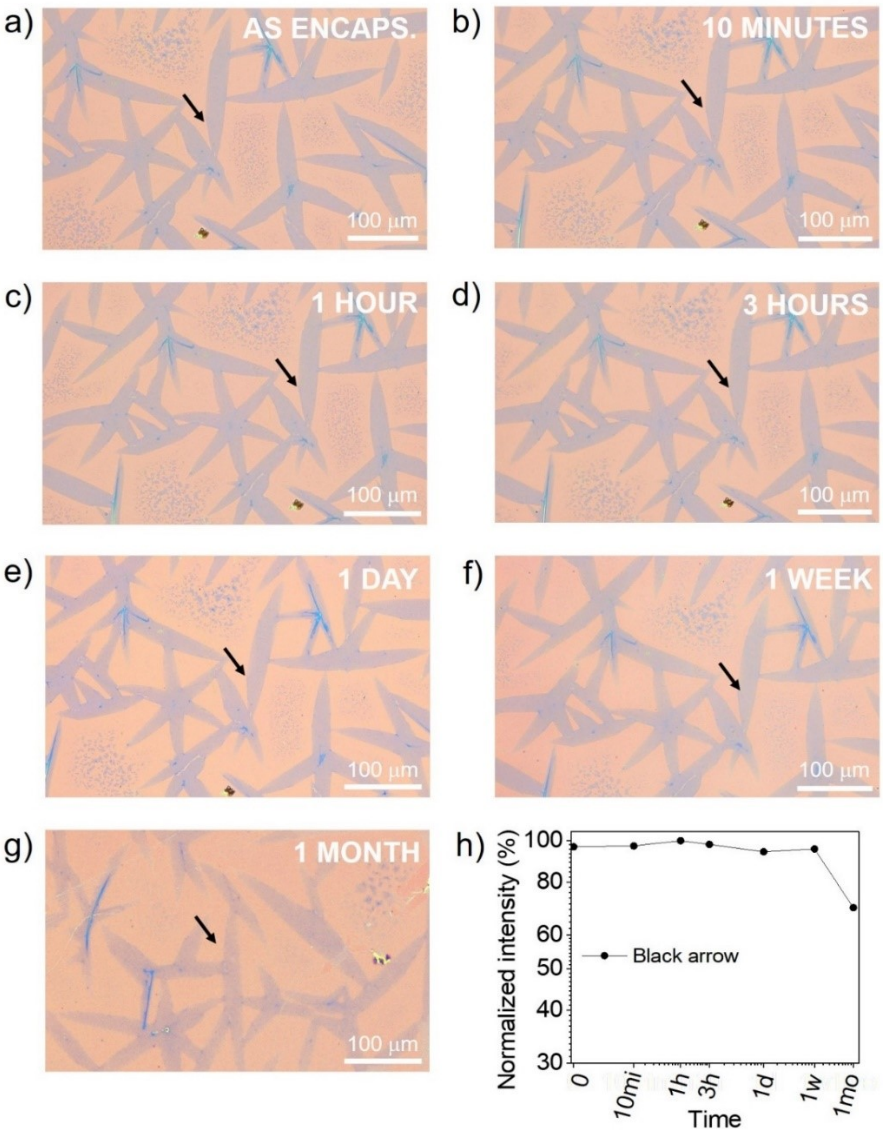

Figure S8 Optical characterization of encapsulated 1T'-MoTe$_2$ at different aging times. Zoom out of the optical images shown in Figure 3d – f of encapsulated 1T'-MoTe$_2$ with CVD hBN right after the encapsulation (a) and 1 hour (b), 3 hours (c), 1 day (d) 1 week (f) and 1 month (g) after the encapsulation. No clear constrast change is visible both for the monolayer (blue contrast) and the multilayer (dark blue contrast) areas within the first 7 days (a – f). After 1 month (g) some fainting of the contrast of the extremities of the crystals starts to be visibile, however the oxidation appears to be prevented on the overall imaged area. h) Quantitative trend of the contrast extrapolated from the area highlighted by the black arrows in panels a – g using the formula $\left(I_{MoTe_2} - I_{Sub}\right)/I_{Sub}$ proposed in ref. 6.

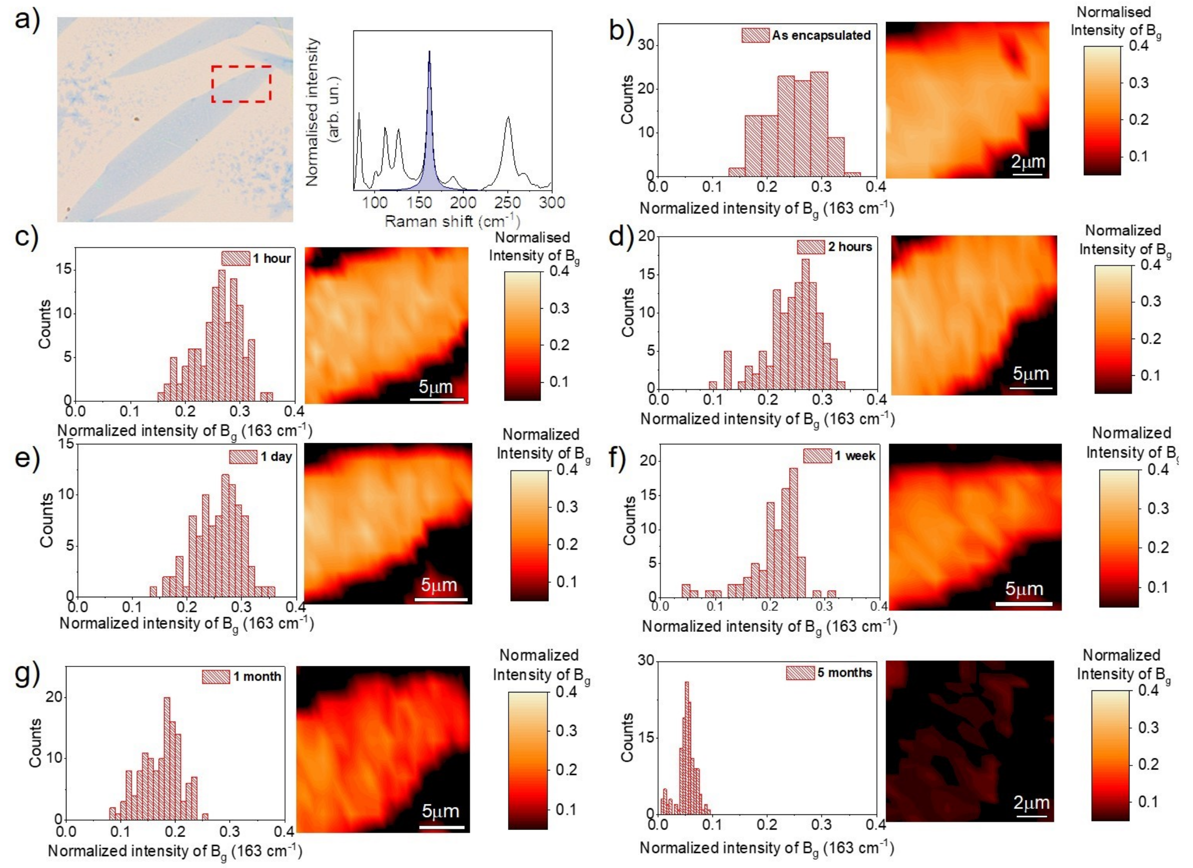

Figure S9 Raman characterization of encapsulated 1T'-MoTe$_2$ at different aging times. a) Optical image and representative single Raman spectrum of encapsulated 1T'-MoTe$_2$. Statistical distribution and Raman maps of the normalized intensity of B_g peak at 163 cm^{-1} (highlighted in blue in panel a) of encapsulated 1T'-MoTe$_2$ right after encapsulation (b) and after 1 hour (c), 2 hours (d), 1 day (e), 1 week (f), 1 month (g) and 5 months (h), taken on the same 1T'-MoTe$_2$ single crystal. The mode of each distribution is used to extrapolate the statistical value of the normalized intensity over time, while the uncertainty is given by the deviation standard. The values extrapolated here are plotted in Figure 4b. To facilitate the comparison of the peak intensity over time, the interval of both *x* axis of statistical distribution and intensity map were fixed between 0 and 0.4 for each aging time. No difference is visible in the normalized intensity of B_g peak for the first 1 week (b – e) while a slow decrease of intensity is visible for the following months, in agreement with optical results shown in Figure S8.

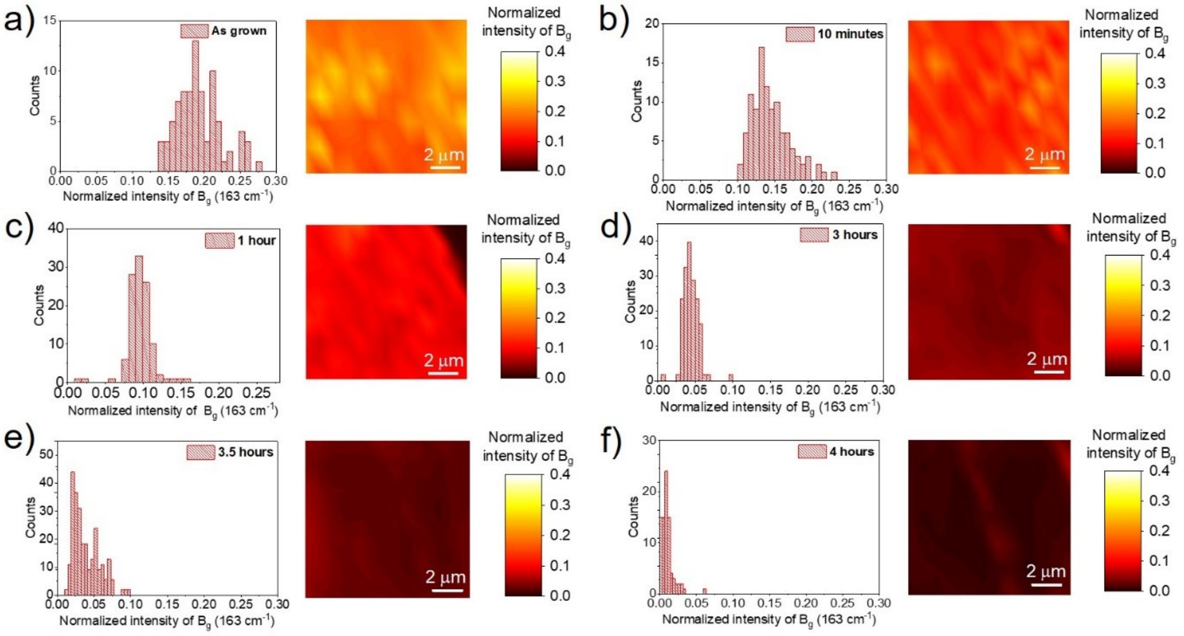

Figure S10 Raman characterization of exposed 1T'-$MoTe_2$ at different aging times. Statistical distribution and Raman maps of the normalized intensity of B_g peak at 163 cm^{-1} of exposed 1T'-$MoTe_2$ right after growth (a) and after 10 minutes (b), 1 hour (c), 3 hours (d), 3 hours and 30 minutes (e) and 4 hours (g), taken on the same 1T'-$MoTe_2$ single crystal. A clear decrease of intensity is visible and approaches 0 within the first 4 hours after the growth, in agreement with the optical results shown in Figure 3a – c) and previous works. [7] The mode of each distribution is used to extrapolate the statistical value of the normalized intensity over time, while the uncertainty is given by the deviation standard. The values extrapolated here are plotted in Figure 4b. To facilitate the comparison of the peak intensity over time, the interval of both *x* axis of statistical distribution and intensity map were fixed at the same values for all panels.

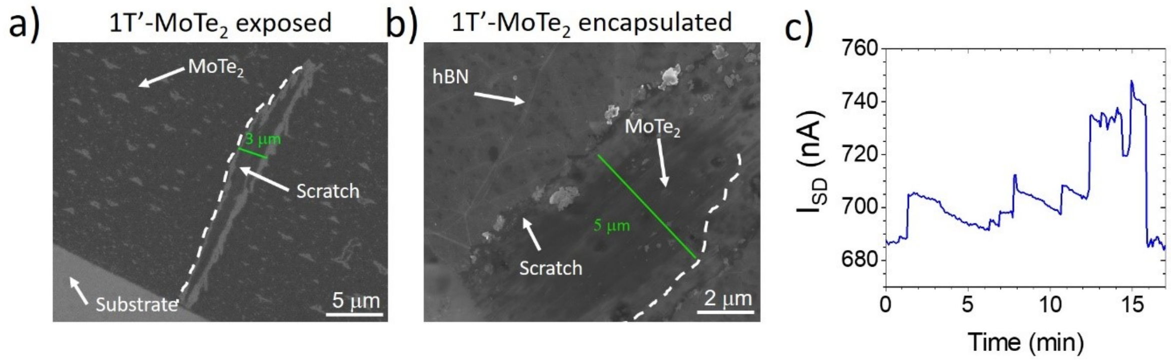

Figure S11 Investigation of the effect of the metal needles on 1T'-$MoTe_2$. a) Image of an exposed 1T'-$MoTe_2$ crystal. After the measurement, the contacted area is visible on the crystal (highlighted by the white dotted line), however the single layer is not completely broken by the needle. b) Detail on a scratched encapsulated crystal shows that the contact area between the metal needle and the crystal is a few micrometers. The coverage of hBN in this area appears to be removed and damaged (dotted line), while the rest of the crystal is still properly covered. The length of the scratch in both panel a and b is due to the mechanical vibrations of the needles during the measurement, which occur along the needle axes, while the lateral size (indicated in green in panel a and b) is strictly related with the contact cross-section of the needle on the sample. c) Example of continuous acquisition of the current flowing in the exposed 1T'-$MoTe_2$ crystal: a constant bias of 0.1 V is applied between the two needles and the current values are recorded every 1 second. The current remains relatively stable for the first 15 minutes of the measurement, within a 5% deviation. The abrupt changes in the measured current are mainly due to mechanical vibrations and micrometric instability of the setup, which might cause the movement of the needles.

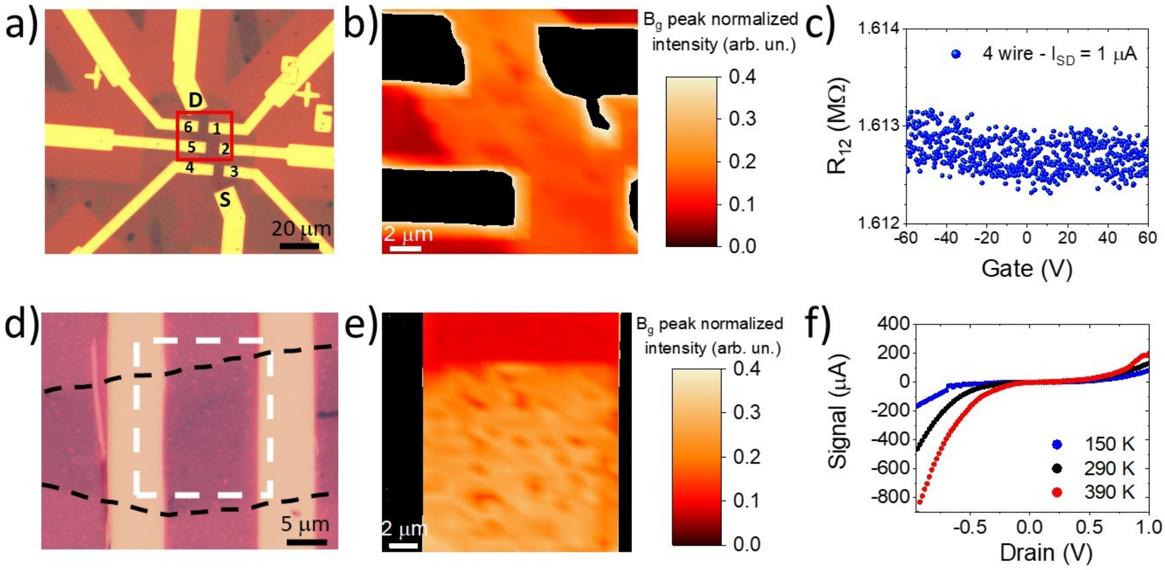

Figure S12 Characterization of fully-fabricated devices on encapsulated 1T'-MoTe$_2$. a) Optical image of an 8-terminal Hall bar realized on encapsulated monolayer 1T'-MoTe$_2$ transferred on a pristine SiO_2/Si substrate. hBN and MoTe$_2$ have been etched away from the area below and around the metal pads to have improved metallic adhesion to the substrate and to avoid multiple single-crystal contacting the same contacts pair. b) Raman map of the normalized intensity of the B_g peak on the Hall bar after full fabrication (taken in the area highlighted by the red square in panel a), confirming that the crystal quality has not been affected by the fabrication process and the Raman intensity is comparable to the pristine one (Figure S9). c) Transfer curve of encapsulated 1T'-MoTe$_2$ single-crystal. By performing a 4-terminal (labelled as D, S, 1 and 2 in panel a) measurement, we only take into consideration the MoTe$_2$ channel resistance. No relevant resistance change was observed in the device as a function of the gate voltage, compatible with a metallic behavior. d) Optical image of a 1T'-MoTe$_2$ device used for temperature-dependent measurements. e) Raman intensity map of the B_g peak, taken in the area highlighted by the white square, after full fabrication of the device and several heating and cooling ramps between 153 K and 393 K (see Methods section for more details), confirming that the material can stand both fabrication and thermal cycles.

f) Temperature-dependent IV characterization of the encapsulated 1T'-$MoTe_2$ based device shown in panel d: the non-linear behavior suggests the presence of a tunneling barrier, due to the dielectric hBN between the metal contacts and the $MoTe_2$. In panel d a relatively weak temperature dependence of the tunneling barrier is also visible, as already reported for hBN from the same source. [8]

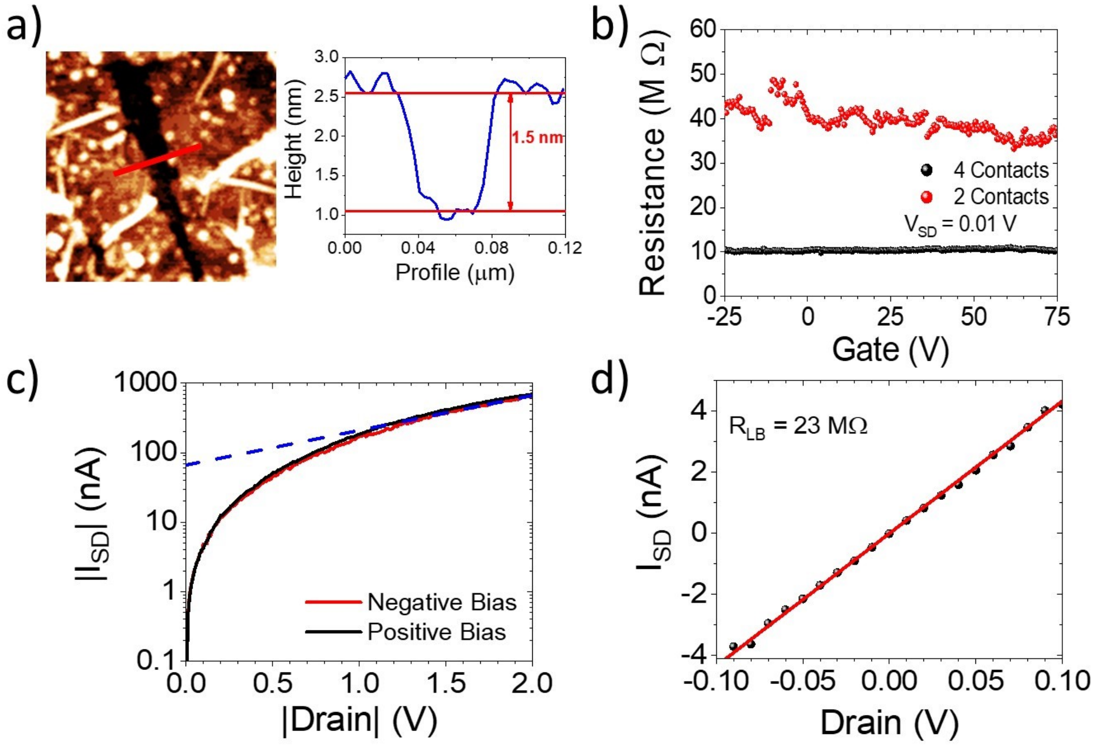

Figure S13 Characterization of the hBN/1T'-MoTe₂ tunneling junction. a) AFM image of a tear in the hBN film and related line profile analysis (red line in AFM image). From line profile analysis the thickness of hBN is equal to 1.5 nm, compatible with 3-4 layers thickness. [9] b - d) Evaluation of the tunneling barrier through commercial hBN film: b) The comparison of resistance in 2-terminal and 4-terminal configuration shows a change in resistance of approximately 30 MΩ. c) Characteristic IV curves for metal/hBN/$MoTe_2$ TLM device in semi-log scale showing an exponential growth for high bias (blue dashed line) in agreement with a tunneling barrier behavior. [8,10] d) Low-bias regime IV curve showing linear behavior and a resistance in the order of 10^7 Ω. Considering as contact area the superposition of the $MoTe_2$ with one metallic contact, the resistance multiplied by the tunneling area is in the order of 10^9 Ω μm^2, similar to the value reported from Britnell and co-workers [11] for 4 layer hBN as well as to other works employing the same *"Graphene-Supermarket"* hBN. [8,12] If the IV curve at low bias in panel d is fitted using the formula reported by Lee and co-workers in ref. 10, assuming a contact area of 100 μm^2 and the thickness of 1.5 nm, a barrier height of 4.9 eV is

obtained, in reasonable agreement with the values reported in ref. 10. The discrepancy of the tunneling barrier value reported here, with respect to the expected one, [10] can be ascribed to the uncertainty in defining the effective contact area, thickness of hBN (Figure S7) and its convolution with the barrier height in the equation describing the IV curve in the low-bias tunneling regime.

.

SUPPLEMENTARY REFERENCES

(1) Wang, J.; Luo, X.; Li, S.; Verzhbitskiy, I.; Zhao, W.; Wang, S.; Quek, S. Y.; Eda, G. Determination of Crystal Axes in Semimetallic T′-$MoTe_2$ by Polarized Raman Spectroscopy. *Adv. Funct. Mater.* **2017**, *27* (14), 1604799. https://doi.org/10.1002/adfm.201604799.

(2) Keum, D. H.; Cho, S.; Kim, J. H.; Choe, D.-H.; Sung, H.-J.; Kan, M.; Kang, H.; Hwang, J.-Y.; Kim, S. W.; Yang, H.; Chang, K. J.; Lee, Y. H. Bandgap Opening in Few-Layered Monoclinic $MoTe_2$. *Nat. Phys.* **2015**, *11* (6), 482–486. https://doi.org/10.1038/nphys3314.

(3) Naylor, C. H.; Parkin, W. M.; Ping, J.; Gao, Z.; Zhou, Y. R.; Kim, Y.; Streller, F.; Carpick, R. W.; Rappe, A. M.; Drndić, M.; Kikkawa, J. M.; Johnson, A. T. C. Monolayer Single-Crystal 1T′-$MoTe_2$ Grown by Chemical Vapor Deposition Exhibits Weak Antilocalization Effect. *Nano Lett.* **2016**, *16* (7), 4297–4304. https://doi.org/10.1021/acs.nanolett.6b01342.

(4) Kim, H.; Han, G. H.; Yun, S. J.; Zhao, J.; Keum, D. H.; Jeong, H. Y.; Ly, T. H.; Jin, Y.; Park, J.-H.; Moon, B. H.; Kim, S.-W.; Lee, Y. H. Role of Alkali Metal Promoter in Enhancing Lateral Growth of Monolayer Transition Metal Dichalcogenides. *Nanotechnology* **2017**, *28* (36), 36LT01. https://doi.org/10.1088/1361-6528/aa7e5e.

(5) Graphene Laboratories Inc. Graphene-Supermarket. https://graphene-supermarket.com/ (Accessed on July 1st 2020).

(6) Naylor, C. H.; Parkin, W. M.; Gao, Z.; Kang, H.; Noyan, M.; Wexler, R. B.; Tan, L. Z.; Kim, Y.; Kehayias, C. E.; Streller, F.; Zhou, Y. R.; Carpick, R.; Luo, Z.; Park, Y. W.; Rappe, A. M.; Drndić, M.; Kikkawa, J. M.; Johnson, A. T. C. Large-Area Synthesis of High-Quality Monolayer 1T'-WTe_2 Flakes. *2D Mater.* **2017**, *4* (2), 021008. https://doi.org/10.1088/2053-1583/aa5921.

(7) Han, G. H.; Keum, D. H.; Zhao, J.; Shin, B. G.; Song, S.; Bae, J. J.; Lee, J.; Kim, J. H.; Kim, H.; Moon, B. H.; Lee, Y. H. Absorption Dichroism of Monolayer 1T′-$MoTe_2$ in Visible Range. *2D Mater.* **2016**, *3* (3), 031010. https://doi.org/10.1088/2053-1583/3/3/031010.

(8) Kamalakar, M. V.; Dankert, A.; Kelly, P. J.; Dash, S. P. Inversion of Spin Signal and Spin Filtering in Ferromagnet|Hexagonal Boron Nitride-Graphene van der Waals Heterostructures. *Sci. Rep.* **2016**, *6* (1), 21168. https://doi.org/10.1038/srep21168.

(9) Li, L. H.; Chen, Y. Atomically Thin Boron Nitride: Unique Properties and Applications. *Adv. Funct. Mater.* **2016**, *26* (16), 2594–2608. https://doi.org/10.1002/adfm.201504606.

(10) Lee, G.-H.; Yu, Y.-J.; Lee, C.; Dean, C.; Shepard, K. L.; Kim, P.; Hone, J. Electron Tunneling through Atomically Flat and Ultrathin Hexagonal Boron Nitride. *Appl. Phys.

Lett. **2011**, *99* (24), 243114. https://doi.org/10.1063/1.3662043.

(11) Britnell, L.; Gorbachev, R. V.; Jalil, R.; Belle, B. D.; Schedin, F.; Katsnelson, M. I.; Eaves, L.; Morozov, S. V.; Mayorov, A. S.; Peres, N. M. R.; Castro Neto, A. H.; Leist, J.; Geim, A. K.; Ponomarenko, L. A.; Novoselov, K. S. Electron Tunneling through Ultrathin Boron Nitride Crystalline Barriers. *Nano Lett.* **2012**, *12* (3), 1707–1710. https://doi.org/10.1021/nl3002205.

(12) Dankert, A.; Venkata Kamalakar, M.; Wajid, A.; Patel, R. S.; Dash, S. P. Tunnel Magnetoresistance with Atomically Thin Two-Dimensional Hexagonal Boron Nitride Barriers. *Nano Res.* **2015**, *8* (4), 1357–1364. https://doi.org/10.1007/s12274-014-0627-4.
